# Memory and Resting‐State Connectivity in Acute Transient Global Amnesia: A Case–Control fMRI Study

**DOI:** 10.1002/acn3.70396

**Published:** 2026-04-10

**Authors:** Elias El Otmani, Emmanuel Barbeau, Béatrice Lemesle, Emilie Milongo‐Rigal, Sophie Fernandez, Sandrine Charpentier, Jean‐François Albucher, Nicolas Raposo, Fabrice Bonneville, Patrice Péran, Jérémie Pariente

**Affiliations:** ^1^ ToNIC Toulouse NeuroImaging Center, UMR 1214, Universit. De Toulouse, INSERM, Paul Sabatier University (UT3) Toulouse France; ^2^ CNRS, Cerco Toulouse France; ^3^ Department of Neurology Neuroscience Centre, Toulouse‐Purpan University Hospital Toulouse Cedex France; ^4^ Department of Emergency Medicine University Hospital of Toulouse Toulouse France; ^5^ Laboratory of Epidemiology and Analyses in Public Health UMR 1295 Inserm, Toulouse University France; ^6^ Department of Neurolog Clinical Investigation Center, CIC1436, Toulouse University Hospital Toulouse France; ^7^ Department of Neuroradiology University Hospital of Toulouse Toulouse France

**Keywords:** episodic memory, memory networks, transient global amnesia

## Abstract

**Background and Objectives:**

Transient global amnesia (TGA) is a striking model of isolated amnesia. While hippocampal lesions are well described, the network‐level mechanisms and the precise neuropsychological profile remain debated. Our objective was thus to characterize functional and neuropsychological correlates of acute TGA and their longitudinal evolution.

**Methods:**

Prospective, single‐center case–control study of 20 patients with acute TGA and 20 age‐ and sex‐matched healthy controls. All participants completed neuropsychological testing and underwent structural and functional MRI at three time points: acute phase (< 24 h from onset), day 3, and 3 months. Primary outcomes were neuropsychological performance across episodic, semantic, and metamemory domains and resting‐state fMRI connectivity within the episodic memory network. Secondary outcomes were functional connectivity within the Default Mode (DMN), Executive (ECN), and Salience (SN) networks.

**Results:**

A total of 40 participants were included (20 patients with TGA, mean age 65.5 years, 45% women; 20 controls, mean age 64.3 years, 45% women). In patients, median delay from symptoms' onset to MRI was 6.67 h. Neuropsychologically, patients showed profound multimodal anterograde amnesia during the acute phase, resolving by 3 months. This deficit was largely isolated, sparing semantic memory and metamemory. Structurally, small bilateral lesions were present in most patients. Functionally, acute hypoconnectivity was observed within the extended hippocampal system, particularly between parahippocampal and cingulate cortices, normalizing by 3 months. No consistent disruption was found in large‐scale networks (default mode, executive control, salience).

**Interpretation:**

TGA is associated with transient, selective hypoconnectivity within the mesiotemporal–cingulate episodic memory network, aligning with previous reports and further precising the functional anatomy. The finding of a profound anterograde amnesia was replicated and its recovery timecourse was elucidated. Semantic memory and metamemory remain preserved, clarifying inconsistencies in prior reports. These findings suggest that TGA reflects a transient limbic dysconnectivity syndrome rather than a diffuse network disorder, reconciling structural lesions with clinical and functional data.

## 1 Introduction

Transient global amnesia (TGA) is defined as the sudden occurrence of anterograde and retrograde amnesia usually lasting less than 24 h, without any other neurological deficits. Diagnosis is based on Hodges' and Warlow's criteria [1]. Proposed etiologies include venous congestion and migraine‐related mechanisms, but neither fully accounts for the syndrome [2].

Neuropsychologically, TGA features marked anterograde episodic amnesia [3] (causing temporal disorientation and repetitive questioning, and required for diagnosis under Kaplan's and Hodges' criteria [1]), and milder retrograde amnesia following Ribot's law, meaning that more recent memories are more likely to be lost than older ones.

The picture is less clear‐cut regarding retrograde semantic memory and/or remote memories. While autobiographical semantic memory is unaffected (importantly for differential diagnosis [1]) and memory of places and object names appears intact [4, 5], mixed results have been found regarding knowledge of historical events [6, 7, 8] or recognition of famous faces [7, 9].

Furthermore, although vocabulary and letter fluency are preserved [10], tests of semantic fluency have repeatedly shown an impairment in TGA [5, 6, 11, 12, 13], both in terms of correct answers and perseverations.

Finally, conflicting reports can be found regarding impairment of metamemory [7, 14].

Procedural memory and visuospatial abilities remain unaffected [7, 15], as well as language [7, 8, 10], executive functions, working memory (WM), and attentional control [6, 7, 10, 11, 14, 15, 16, 17, 18], although subtle opposite evidence has occasionally been found [19]. See Liampas et al. [20] for a recent review.

High‐resolution MRI reveals small CA1 hippocampal lesions detectable up to 13 days post‐onset in 80% of patients, bilateral in ~10% of cases, and resolving within 2 weeks [21]. Their delayed detection and small size make their relationship to severe acute amnesia unclear.

Conversely, recent resting‐state functional imaging studies carried in the acute phase have tackled the hypothesis of TGA as a network disease. Using fMRI, Peer et al. [22] showed decreased functional connectivity in 12 patients relative to controls within a set of regions overlapping with most of the extended hippocampal system, and which they called the Episodic Memory Network (EMN). Similarly, Segobin et al. [23] conducted a Positron Emission Tomography study on 10 patients, revealing disrupted group‐level metabolic correlations within the limbic circuit.

Regarding major resting‐state networks, an fMRI study of 16 patients [24] showed decreased functional connectivity within the Executive Control Network (ECN) as assessed through Independent Component Analysis (ICA), whereas a similar work in 50 patients found functional connectivity to be unchanged in the ECN but increased in the Salience Network (SN) and decreased in the Default Mode Network (DMN), with normalization at three months [25]. These findings are difficult to interpret in light of the severe and seemingly isolated amnesic syndrome observed in patients with TGA.

The present study provides a novel evaluation of acute‐phase and recovery timecourses of resting state connectivity disruptions and neuropsychological impairments in 20 patients matched with 20 controls, testing the hypothesis that TGA is primarily a disease of the extended hippocampal system and, correspondingly, of hippocampus‐dependent memory. fMRI was conducted at a median delay of 6.67 h hours after symptom onset, and again at 3 days and 3 months, each session preceded by neuropsychological testing.

## 2 Materials and Methods

### 2.1 Participants

From April 2, 2014 to June 8, 2015 we prospectively recruited patients admitted to Toulouse University Hospital's emergency department for ongoing TGA, diagnosed according to Hodges' criteria [1]: (i) witnessed anterograde amnesia; (ii) isolated amnesia without altered consciousness or loss of identity; (iii) absence of focal neurological/epileptic signs or recent head trauma. Patients were retrospectively excluded if amnesia persisted for longer than 24 h. None had a psychiatric or neurological history except prior TGA episodes. Healthy controls were concurrently recruited, matched for age, gender, and education. As this was a biomedical study in an emergency setting, patients could be included upon arrival at the emergency department without prior consent. When possible, consent was obtained from a relative or accompanying person in accordance with French law (Article L.1122‐1‐2). In all cases, informed consent from the patient was systematically collected at 72 h once recovery allowed. Control subjects gave written informed consent at the first visit. The protocol was approved by the local ethics committee (2013‐A01271‐44). See Supplementary Methods for details on inclusion and exclusion criteria.

### 2.2 General Study Design

**FIGURE 1:**
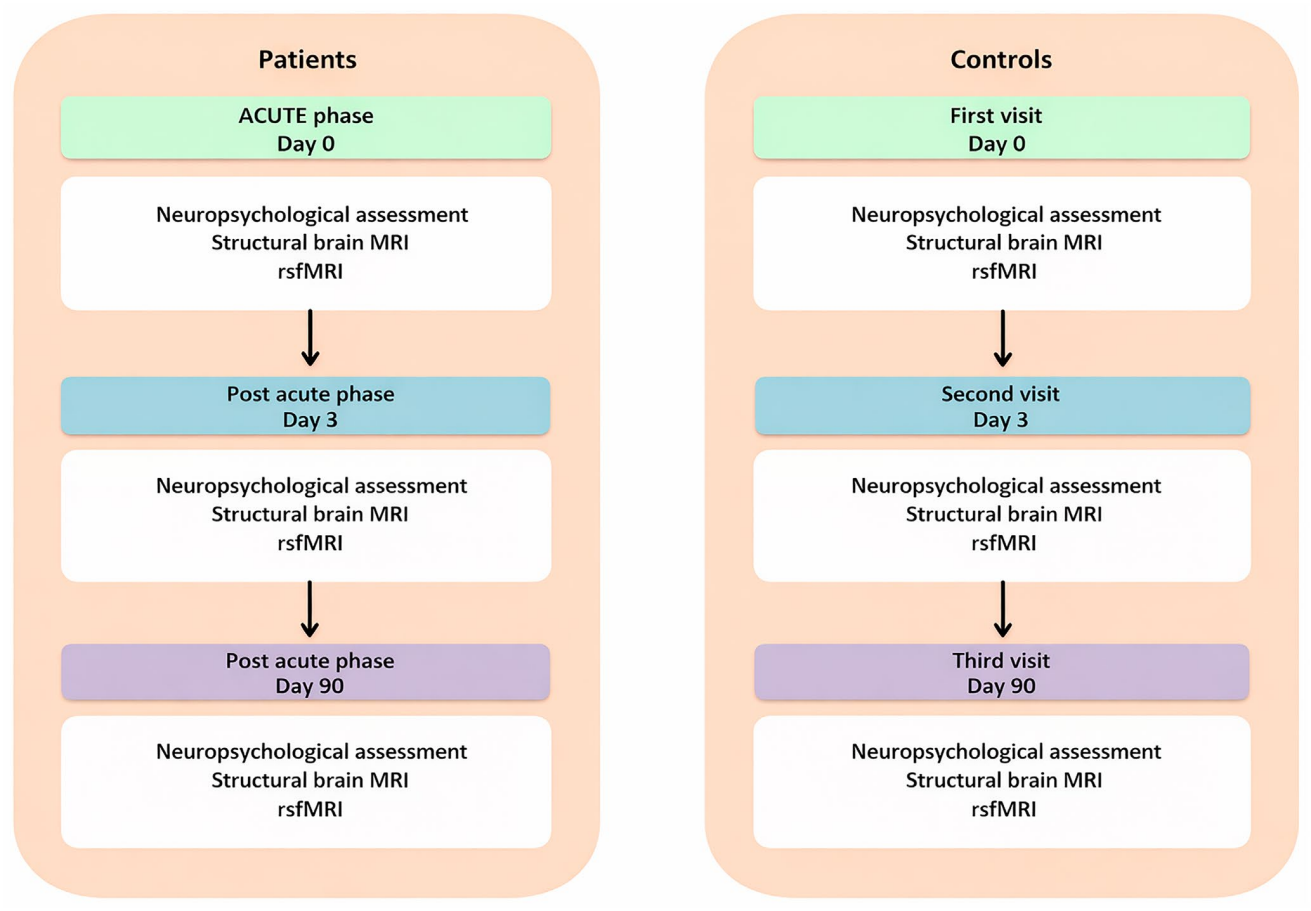
Study design.

**FIGURE 2:**
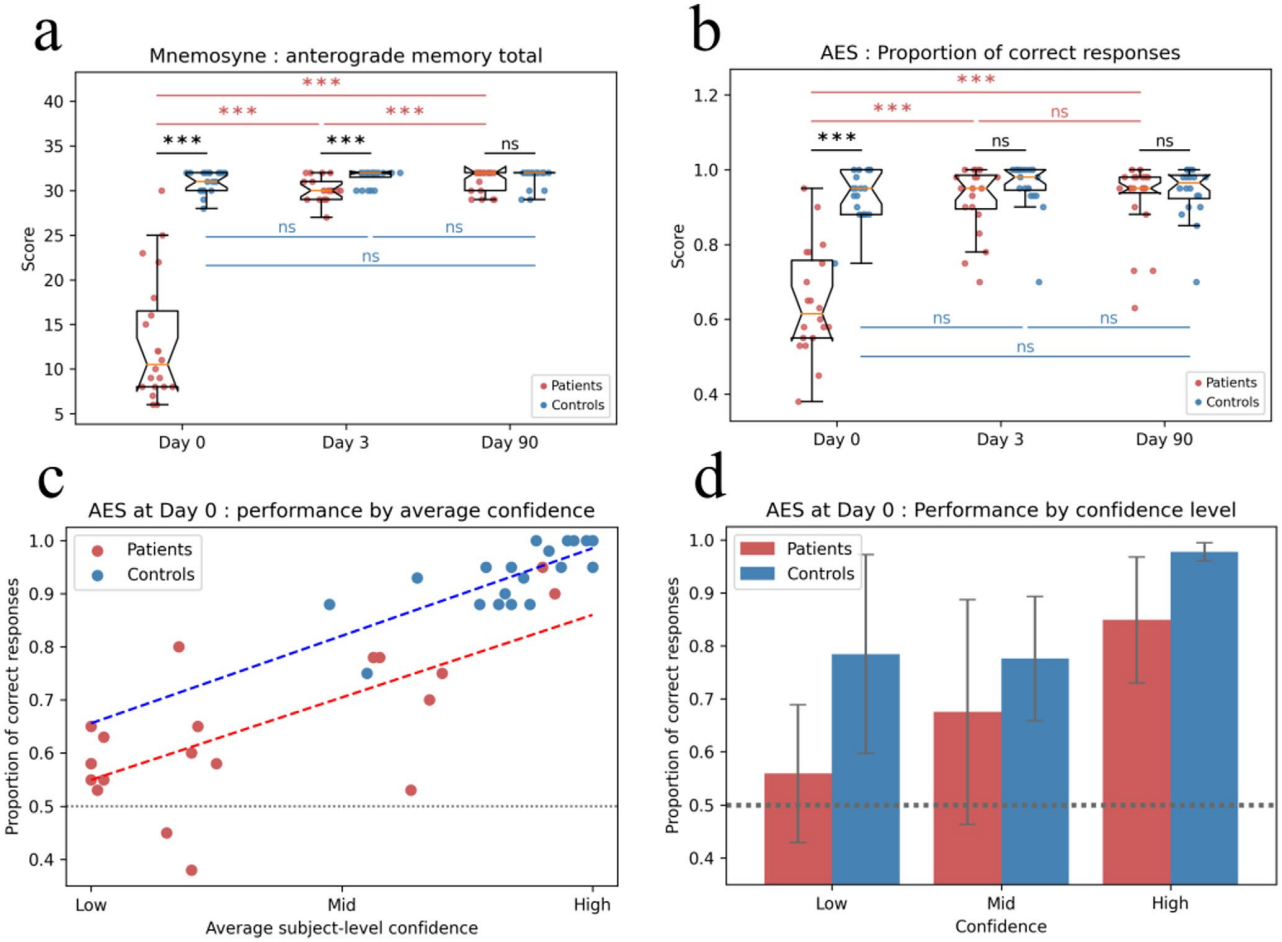
Neuropsychological evaluation: Anterograde memory and metamemory. Box notches indicate 95% confidence intervals. Symbols “ns”, “*”, “**” and “***” respectively indicate a *p* > 0.05, < 0.05, < 0.01, < 0.001. (a) Anterograde memory component of Mnemosyne evaluation. (b) Proportion of correct responses in the anterograde memory AES test. (c) AES test performance as a function of the subject‐average confidence (each dot is one subject). Red and blue dashed lines: Least‐squares regression lines for Patients and Controls, respectively. Gray dotted line: Chance level. *ρ*: Spearman's correlations. (d) AES test performance for each level of confidence. Gray dotted line: Chance level. Error bars: 95%‐level confidence intervals.

Patients were assessed at Day 0 (acute phase), Day 3, and Day 90 post‐onset; controls underwent the same three sessions. Each included neurological and neuropsychological testing, structural MRI, and resting‐state fMRI (Figure 1). Acute TGA was defined as Dubois test [26] ≤ 8/10 and/or a Mini‐Mental State Examination (MMSE) [27] orientation score ≤ 7/10, assessed before (inclusion criteria) and right after MRI (Figure 2).

### 2.3 Neuropsychological Battery

We developed the Mnemosyne neuropsychological evaluation (Béatrice Lemesle & Emmanuel Barbeau), meant to evaluate many different aspects of memory in a time‐efficient manner. It comprises three parts. First, a prospective memory task: participants must clap their hands three times when a 4‐min timer rings. In case of failure, three successive cues are provided: a general reminder, a hint involving their hands, and finally the explicit instruction to clap. Second, an incidental anterograde memory task: four unnoticed “mini‐events” are presented—viewing a photo of a famous event, rolling a die, using a tape measure, and smelling two odors. After 4 min, memory is tested by free recall, cued recall, then recognition. If an event is recalled freely, later tests are omitted and full credit is given; if recalled with a cue, recognition is skipped.

The anterograde memory total score (32 points) combines these two sections. Third, a retrograde memory task: participants identify a photo of the September 11 attacks first in free recall, then in a three‐choice recognition test. After being told the correct answer, they recall the reason for the attacks and a famous person involved. These performances form the retrograde memory total (10 points). Finally, the episodicity of their 9/11 memory is rated separately with the TEMPau scale [28], based on whether they recalled what they were doing when the events occurred.

We also used AES [29] (French acronym of Animals In Scenes), a computerized visual recognition test that implied memorization of 40 animal–scene pictures, later presented in a series of two‐possibilities forced‐choice recognition tasks after a 1‐min distractor. Total score was the proportion of correct responses (chance level: 50%). Participants gave choicewise confidence ratings (“Low”, “Mid”, “High”).

Other tests included two subsets of the Weschler Adult Intelligence Scale (WAIS) III [30]: Information (semantic memory) and Digit‐Symbol Coding subset (general cognitive function) [31]. We also tested verbal fluency over 1 min (“syntactic”: letter “P”, and semantic: animals), picture denomination (10 pictures from the DO80 [32]) only at session 1, orientation (from the MMSE), and the State–Trait Anxiety Inventory (STAI) [33].

Except for DO80, tests were assessed for all participants at all three timepoints. Tests were chosen to be brief and avoid delaying MRI.

### 2.4 MRI

See **Supplementary Methods** for details on the MRI acquisition, preprocessing, and analysis methods.

For hippocampal lesion assessment, a blinded neuroradiologist (FB) assessed hippocampal DWI and T2 hyperintensities. Lesions were mapped according to Duvernoy's atlas [34], segmented, and coregistered to the Montreal Neurological Institute (MNI) template.

fMRI data preprocessing included realignment, slice timing correction, outlier detection, direct segmentation, MNI‐space normalization, smoothing and denoising.

For EMN determination we used a network evaluated in Peer et al.'s study [22], which was based on a meta‐analysis of 24 functional imaging studies outlining the brain regions involved in episodic memory [35]. The 15 corresponding ROIs were selected from the Automatic Anatomical Labeling atlas 3 [36]. We then computed ROI‐to‐ROI connectivity matrices (see Supplementary).

SN, ECN and DMN, as major resting‐state networks, were extracted and assessed with ICA (see Data S1).

### 2.5 Statistical Analysis

For demographics, Fisher's exact or Mann–Whitney tests compared groups.

For neuropsychological scores, two‐way repeated‐measures ANOVA (Time × Group) was used. Non‐normal data (Shapiro *p* < 0.05) were first transformed via Aligned Rank Transform (ART) [37]. Post hoc *t*‐tests were run between groups (if group effect; independent samples) and between sessions within groups (if time effect; repeated‐measures) with a *p* < 0.05 threshold.

In EMN second‐level analyses, each ROI was treated as a cluster (all its connections), with a separate General Linear Model (GLM) [38] estimating connection‐level connectivities as dependent variables and group/session as predictors. ROI‐level hypotheses were tested using multivariate parametric statistics with random effects across subjects and covariance estimation across repeated measures. Inferences were made at the cluster level with familywise correction (p‐FWE < 0.05). For significant clusters, connection‐level GLMs were then fitted to identify the connections driving the effect; post hoc significance was defined as uncorrected *p* < 0.01.

Each participant's whole‐network EMN connectivity was the mean of all its connectivity values. It was analyzed through mixed model ANOVA.

Second‐level analyses of DMN, ECN, and SN were conducted using voxelwise GLMs, with first‐level connectivity as dependent variables and groups/sessions as predictors. Random‐effects and covariance across sessions were modeled, allowing cluster‐level inferences using Gaussian Random Field theory (cluster‐forming voxel threshold of *p* < 0.001, cluster‐size correction at *p*‐FDR < 0.05).

## 3 Results

### 3.1 Population

We included 20 acute‐phase TGA patients and 20 matched controls (Table 1); flow diagram in Figure S1. Median delay between onset and MRI was 6.67 h (mean: 10.68 h); in two cases, onset time was unknown. Twelve of 19 retested post‐MRI still met the acute TGA definition (Dubois' score ≤ 8/10 and/or MMSE orientation ≤ 7/10).

**TABLE 1:** Participants' demographics, medical history and precipitating factors.

Criterion	Patients (*n* = 20)	Controls (*n* = 20)	*p* (*t*‐test)	*p* (Mann–Whitney U)
Matching criteria				
Age	65.55	63.4	0.28	
Females: *n*, (%)	11 (45%)	11 (45%)	1	
Education (mean study years since 1st grade)	11.3	11.7	0.72	0.571
Medical history: *n*, (%)*				
TGA	2 (10%)	0	0.16	0.162
Migraine	0 (0%)	5 (25%)	0.01	0.019
Migraine with aura	1 (5%)	0	0.32	0.345
Epilepsy	0	0		
Stroke	0	0		
Cognitive complaint	1 (5%)	0	0.32	0.343
Hypertension	7 (37%)	3 (17%)	0.18	0.179
Diabetes	0	16%	0.07	0.08
Dyslipidemia	4 (21%)	6 (32%)	0.47	0.479
Tobacco smoking	2 (10%)	2 (11%)	0.95	0.977
Overweight	0	0		
Depressive syndrome	3 (15%)	0	0.07	0.08
Current TGA episode precipitating factors over the last 24 (and 48) hours				
Physical activity	1 (4)			
Emotional shock	2 (4)			
Sexual intercourse	4 (5)			
Exposure to cold water	2 (2)			
Acute pain	0 (1)			

* Percentages occasionally exceed their expected 𝑛/20 proportions because they are calculated based on the actual sample size for each parameter, excluding any missing values.

Groups did not differ in neurological, cardiovascular, or psychiatric history, nor in trait anxiety. Migraine history was more frequent in controls (U = 133.0, *p* = 0.019).

### 3.2 Neuropsychological Results

Unless stated otherwise, tests involved mixed model ANOVA on data transformed through ART (ART‐ANOVA).

Anterograde memory tests showed significant group × time interactions both for Mnemosyne's anterograde subset (Figure 2a) and for AES image recognition test (Figure 2b, both *p* < 0.001). Patients were impaired acutely vs. controls, and at Day 3 for Mnemosyne only, with recovery by Day 90. They showed significant improvement between acute phase at later sessions. Day 3 patient Mnemosyne scores remained below Day 0 controls (*p* = 0.04). Subtest analyses showed deficits in free recall, cued recall, and recognition (Figure S2) and across intentional/incidental learning and visual, auditory, and olfactory modalities. Performance correlated across all anterograde tests (Figure S2).

AES confidence ratings (collected after each forced‐choice) correlated with accuracy (patients: ρ = 0.54, *p* = 0.01; controls: ρ = 0.79, *p* < 0.001, Figure 2c). Patients' performances across their three self‐reported confidence levels showed a significant confidence effect (Kruskal‐Wallis test, *p* = 0.006), indicating accurate item‐level monitoring (Figure 2d).

**FIGURE 3:**
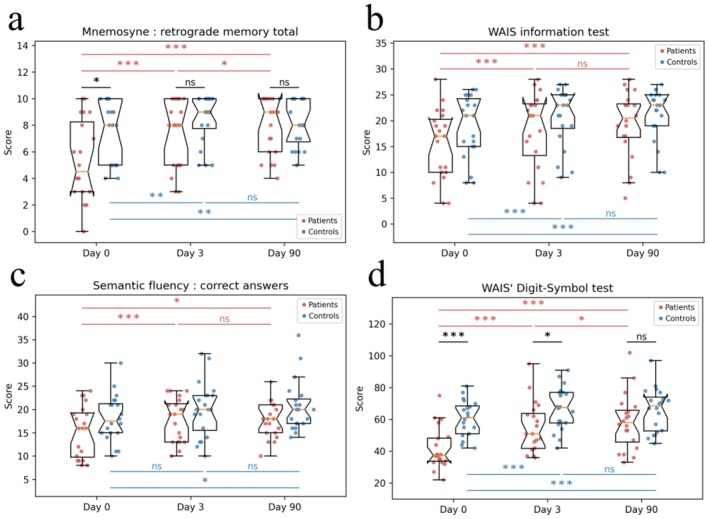
Neuropsychological evaluation: Other tests. Box notches indicate 95% confidence intervals. Significance results of pairwise *t*‐tests are only depicted if the corresponding Group, Time or Interaction factor is found significant in the ART‐ANOVA analysis (no significant Group effect in plot (d)). Symbols “ns”, “*”, “**” and “***” respectively indicate a *p* > 0.05, < 0.05, < 0.01, < 0.001. (a) Retrograde memory component of Mnemosyne evaluation. (b) Information component of the WAIS‐III battery (semantic memory). (c) Semantic fluencies: Number of different animals given in 1 min. (d) Digit‐Symbol Coding component of the WAIS‐III evaluation.

Patients' retrograde memory was also impaired, as Mnemosyne 9/11 scores showed group × time interactions (*p* = 0.011) with trajectories and intergroup differences similar to anterograde memory scores (Figure 3a). Impairments included free recall of the event, dating, and naming key figures, though recognition (out of three possibilities) was preserved in 18/20 cases. TEMPau episodicity loss was non‐significant (interaction *p* = 0.18, Figure S5). WAIS‐III “Information” test of general knowledge about the world showed a time effect (*p* < 0.001), with both groups improving, but no group (*p* = 0.17) nor interaction (*p* = 0.45) effect (Figure 3b). Picture naming was intact (sum of patients' scores: 199/200), and so was smell discrimination.

Fluency tests showed no interaction effects for correct responses (Animals: *p* = 0.90; Letter: *p* = 0.50) or rule errors, but significant effects for repetitions (both *p* < 0.001), driven by acute‐phase Patient's impairments (Figure 3c). WAIS digit‐symbol test revealed group × time interactions (*p* < 0.001), with intergroup differences at Day 0 and 3 and significant intersession improvements in patients (Figure 3d). We also found interaction effects for the STAI‐state anxiety (F = 3.99, *p* = 0.02), which was higher acutely (Figure S4).

### 3.3 Structural MRI

**FIGURE 4:**
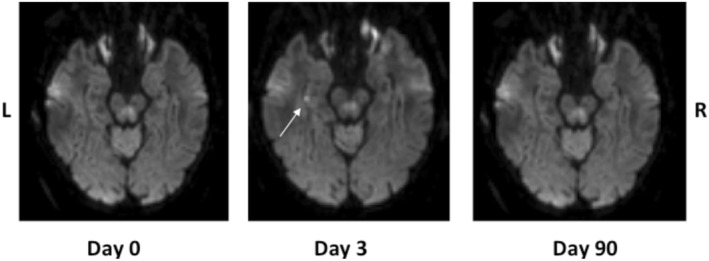
Evolution of a typical hippocampal lesion in one patient. Day 0: Initial brain MRI performed with high‐resolution diffusion‐weighted imaging during the acute phase is normal. Day 3: High‐resolution diffusion‐weighted imaging obtained 3 days after symptom onset shows a small, round, hyperintense lesion in the right hippocampus (arrow), which was no longer visible 90 days after onset on Day 90.

At Day 0, 10 patients (50%) had hippocampal lesions; they were 18 (90%) at Day 3. Patients had between 0 and 4 lesions (mean = 1.4). Lesions (*n* = 29) were mostly in CA1 (83%), left‐lateralized (55%), and in the hippocampus' body (55%‐ vs. 24.1% tail and 20.7% head). See Figure 4 for a typical example. When multiple (7 patients), lesions were diffuse (no two lesions landing in the same hippocampal division), and mostly bilateral and symmetrical (Table S1).

No lesions persisted at Day 90. No other DWI lesions (in particular ischaemic) were found. Controls had normal MRI.

### 3.4 Functional MRI

**FIGURE 5:**
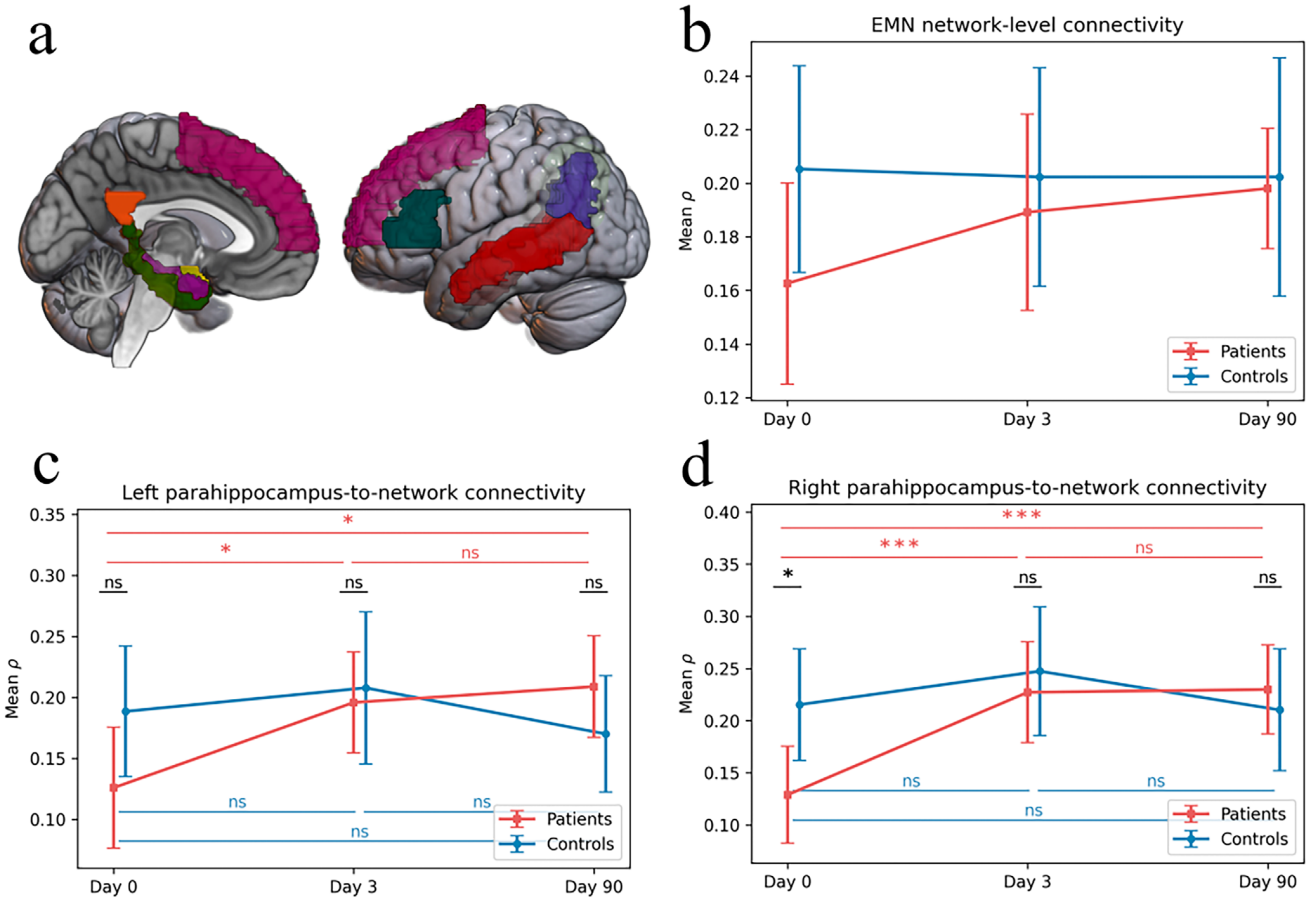
Resting‐state connectivity of the Episodic Memory Network. Brackets indicate 95%‐level confidence intervals. Significance results of pairwise *t*‐tests are only depicted if the corresponding factor (Group, Time, Interaction) is found significant in the ART‐ANOVA analysis (no significant effect in plot (b)). Symbols “ns”, “*”, “**” and “***” respectively indicate a *p* > 0.05, < 0.05, < 0.01, < 0.001. (a) EMN ROIs emphasized on a medial (left) and lateral (right) view of the left hemisphere. They are symmetrical between hemispheres with the exception of right angular gyrus which is not included in the EMN. Pink: Superior frontal gyrus, dark green: Triangular part of the frontal inferior gyrus, blue: Angular gyrus, orange: PCC, light green: PHG, purple: Hippocampus, yellow: Amygala. (b) Network‐level average intrinsic connectivity of the EMN. (c), (d) Average connectivity of the left (c) and right (d) PHG to the rest of the EMN.

EMN's whole‐network mean connectivity (Figure 5a) (average ROI‐to‐ROI correlation) showed a trend towards hypoconnectivity in patients at Day 0, but not at later sessions (Figure 5b), although not reaching statistical significance (*p* = 0.17). At a finer spatial scale, ROI‐level analyses identified three significant connection clusters, corresponding to the left parahippocampal gyrus (PHG) (p‐FWE = 0.006), right PHG (p‐FWE = 0.008), and left posterior cingulate cortex (PCC) (p‐FWE = 0.005). Mean connectivity of either PHG to the rest of the network was significantly reduced only in acute‐phase patients and normalized over time (interaction effects—left: *p* = 0.026; right: *p* = 0.091) (Figure 5c,d). No significant interaction was observed for right PCC (*p* = 0.23). The connections driving these effects linked each PHG to bilateral posterior cingulate, left angular, and left midtemporal cortices (see Supplementary Statistics).

In patients, left and right parahippocampal‐to‐network connectivities were strongly correlated (*ρ* = 0.86, *p* < 0.001), indicating a largely symmetric effect at the individual level. Regarding other networks, ICA identified several components partially overlapping with canonical resting state networks, based on their spatial correlation coefficients (*r*) with the CONN software's default templates [39]. The best matches were selected for ventral DMN (*r* = 0.44), dorsal DMN (*r* = 0.30), and ECN (*r* = 0.42). No strong match was found for the SN, but two components (*r* = 0.27 and *r* = 0.28) were interpreted as its left and right hemispheric subdivisions (Figure S8). No voxel cluster showed significant effects for these maps, except for a significant group × time interaction in a 218‐voxel cluster in the right SN, located in the superior right lateral occipital cortex, reflecting higher patients' connectivity values at sessions 2 and 3.

Linear mixed‐effect model analysis showed no relations between each hippocampal division's (anterior, mid, posterior) Day 0 connectivity and the presence of corresponding structural MRI lesions. There were no significant correlations between fMRI hypoconnectivity in patients (as assessed by their Day 0—Day 90 parahippocampal‐to‐network connectivity differences) and number nor localization of structural MRI lesions, neuropsychological tests, nor delay from symptoms' onset to MRI. No significant correlations were found between the number, localization, bilateral nature, or cumulative volume of hippocampal lesions and anterograde memory impairments (see Figures S6, Supplementary Statistics and Section II‐4 of the online code for details).

## 4 Discussion

Our neuropsychological results confirm the profound anterograde declarative amnesia described in the literature, affecting recall and recognition across multiple encoding modalities. Contrastingly, semantic memory and verbal fluency were largely preserved, and patients retained accurate metamemory at the response level. Structurally, we replicated the presence of small CA1 lesions, of which frequency, timecourses and localization were in general agreement with the literature [40]. Functionally, fMRI revealed transient hypoconnectivity of PHG, especially to PCC. Together, these findings point to a brief, selective dysfunction of the extended hippocampal system.

Performance on the WAIS's Information subtest, which assesses semantic memory, was preserved. This results aligns with previous findings suggesting preserved intact semantic memory in TGA [4, 5]. Similarly, our findings on semantic fluency—no deficit in correct responses despite increased repetitions—casts doubt upon the idea that TGA broadly impairs semantic retrieval [20], based on opposite findings on smaller samples [13]. Most of these studies didn't use “Letter” fluency tests, making it difficult to isolate semantic retrieval from other cognitive processes. Importantly, we observed no increase in category errors, further supporting intact semantic memory.

In contrast, we replicated deficits in recalling and dating public events (e.g., the September 11 attacks), a finding inconsistently reported in previous studies [6, 7, 8]. This dissociation between preserved semantic memory and impaired public‐event memory is informative, as such memories occupy an intermediate position between semantic and episodic domains—combining factual knowledge with the contextual and temporal features of personal experience. Their retrieval likely engages not only semantic stores but also hippocampal–posterior cingulate networks supporting contextual reinstatement and temporal anchoring [41]. However, performance in the corresponding episodic question (what participants were doing when the events unfolded) didn't show significant group nor interaction effects, although a trend towards impaired performances in acute‐phase patients (with significant improvement across sessions) was observed (see Figure S5). The selective deficit for relatively recent public events may also reflect Ribot's law, as the reference event occurred only 14–15 years before testing. Taken together, our results reinforce models positing that semantic memory is independent from the medial temporal lobe [42], yet they don't distinguish between them regarding episodic memory impairment.

Patients' confidence ratings closely tracked actual recognition performance, demonstrating preserved item‐level metamemory. Previous reports suggested either preserved awareness of memory deficits [7] or a more generalized awareness (causing anxious perplexity) without precise insight [14]. These contradictions suggest that metamemory is not unitary: patients may be unaware of encoding deficits but can accurately monitor retrieval. This contrasts with other memory conditions such as Korsakoff's syndrome, in which patients typically display intrusions and confabulations during free recall tasks.

Patients were impaired in WAIS Digit‐Symbol Coding. Although the task partly relies on recalling digit–symbol associations, WM and binding processes [11, 17, 18] as well as processing speed [20] should be preserved. Impairment might reflect anterograde long‐term memory deficit, since WM retains information for ~10 s without rehearsal [43] whereas Digit‐Symbol Coding lasts 1 min. Similar long‐term memory demands may underlie the higher number of repeats in Fluency tests, despite unchanged total scores; yet repeats did not significantly correlate with Digit‐Symbol Coding in our data (Figure S3). As this test is a highly sensitive but nonspecific marker of cognitive dysfunction [31], we do not interpret these results as executive or visuo‐praxic deficits.

Patients' anterograde memory being impaired both for Mnemosyne (recall memory) and AES (recognition memory) tests is compatible with parahippocampal connectivity disruptions, as recognition is thought to rely on subhippocampal (rather than hippocampal) structures [44]. Day 3 recognition performances were fully restored for AES, while residual recollection impairments subsisted for Mnemosyne, supporting the hypothesis of a faster recognition memory recovery [5], although a simpler explanation based on task difficulty cannot be excluded. No differences were found at 3 months for any of the tests, supporting complete recovery. Regarding other medial temporal lobe functions, we found preserved smell discrimination. Studying navigation and place recognition could be an interesting research avenue.

While we did not observe direct correlations between mesiotemporal dysconnectivity (particularly with the PCC) and neuropsychological performances, this finding aligns with Peer et al.'s EMN hypoconnectivity [22], refining the disruption to mesiotemporal–cingular structures despite non‐significant whole‐network results, and with Segobin et al., who described a “fragmentation” of limbic metabolic correlations into a medial temporal cluster and a cingulate–prefrontal cluster [23]. Parahippocampal–cingulate connections, part of the historical Papez’ circuit and the modern extended hippocampal system, are crucial for anterograde memory [45]. Yet parahippocampal links to left angular and middle temporal gyri also contributed, suggesting a broader parahippocampal dysfunction rather than focal disconnection. Importantly, the anterior thalamic nuclei, which are a major part of this extended hippocampal system, were not included in the network we selected from Peer et al. [22]. Therefore, we included them in a supplementary analysis on the extended hippocampal system, which didn't show any significant supplementary effect of this additional structure's connectivity (but see Figure S7).

Connectivity impairment appeared bilateral, consistent with patients' memory loss—deeper than in unilateral hippocampal stroke, affecting both verbal and non‐verbal domains despite hippocampal lateralization [46]. This supports bilateral aetiologies (migraine, metabolic, venous mechanisms) over arterial causes. Note that, Although migraine prevalence appeared higher in controls than in patients, this finding likely represents a false positive, as it is inconsistent with evidence from larger cohorts [47].

Notably, hippocampal dysconnectivity was absent; since PHG are the hippocampus' main cortical gateway [45], this likely reflects limitations in detecting hippocampal disruptions, possibly owing to medial temporal fMRI dropout or specific connectivity dynamics. As in Peer et al. [22], disruptions were acute‐phase only, resolving with memory recovery.

At the network level, we found no significant effects in DMN, ECN, or SN. In contrast, Kim et al. [25] reported DMN decreases in acute TGA, though of unclear significance; differences may reflect our lower statistical power. Still, our EMN results are compatible, given DMN–EMN overlap in mesiotemporal and PCC, suggesting DMN effects may stem from limbic hypofunction. We did not replicate their SN increases (apart from a few questionable occipital voxels), although it is important to consider that, contrary to DMNv and ECN (spatial correlations > 0.4), no acceptable SN component fit was obtained (correlations < 0.3). Finally, we did not replicate ECN decreases [24, 25]. Together with TGA's isolated amnestic syndrome, our results support TGA as a model of selective limbic dysfunction.

Our study has limitations. First, although adequate for an acute TGA cohort, the sample size remains relatively small for an fMRI investigation. This may partly explain why, unlike Peer et al., our effects reached significance only at the ROI level and not at the whole‐network level, despite the strong concordance of the underlying connectivity pattern. Second, our main target, the EMN, is a relatively restricted network. The limited effect size may have contributed to the absence of detectable DMN disruption—expected given the anatomical overlap between the two networks—which might have been captured by a whole‐brain ICA approach that makes fewer assumptions than ROI‐based analyses. Nevertheless, defining the network a priori from Peer et al.'s regions likely reduced the risk of false positives inherent to broader exploratory methods. Third, our findings cannot be interpreted as evidence of a causal mechanism for TGA. On the one hand, they are purely correlational; on the other hand, they pertain only to the recovery phase of the syndrome—that is, downstream of the pathophysiological event that triggered the amnesia—despite our efforts to minimize the delay between symptom onset and MRI acquisition. Finally, the lack of significant correlations between connectivity measures and memory performance constrains the interpretability of our findings, possibly due to floor effects in the neuropsychological scores.

## 5 Conclusion

Our results further establish TGA as a profound, modality‐independent, verbal and non‐verbal isolated declarative memory impairment. Moreover, we show that the retrograde component of this dysfunction largely spares semantic memory. We also replicate previous findings of disrupted functional connectivity between regions involved in episodic memory and provide the first evidence that this effect is bilateral, primarily affecting the PHG and its connections with the PCC. Taken together, our neuropsychological and fMRI findings strongly support the notion of an isolated bilateral medial temporal lobe dysfunction. Further studies are needed to clarify the mechanisms underlying this transient impairment.

## Author Contributions

J.P. and P.P. designed and supervised the study, and acquired funding. E.B. and B.L. designed the neuropsychological protocol. E.M.‐R., S.F., S.C, J.‐F.A., and N.R. recruited the patients. B.L. and E.M.‐R. conducted the neuropsychological assessments. E. E., P.P., F.B., and E.M.‐R. analyzed the MRI data. E.E., E.B., B.L., and E.M.‐R. analyzed the neuropsychological data. E.E. and E.M.‐R. performed the literature review. E.E. drafted the manuscript. J.P., P.P., E.B., and B.L. critically reviewed and edited the manuscript.

## Funding

This work received funding from Institut des Sciences et du Cerveau de Toulouse (TMBI). CHU de Toulouse. Université Paul Sabatier.

## Conflicts of Interest

The authors declare no conflicts of interest.

## Supporting information


**Figure S1:** Patients' flow diagram.
**Figure S2:** Total free recall, cued recall and recognition performances in Mnemosyne's episode 2 at day 0. This section of the Mnemosyne evaluation is a multimodal incidental encoding task.
**Figure S3:** Correlation matrix and clustering dendrogram of patient's performances in neuropsychological testing at Day 0. “Who” and “What” refer to questions about the 9/11 attack, asking for recall of, respectively, a famous character involved and the reason for the attack. Other tests are detailed in the Methods section.
**Figure S4:** state of anxiety between Patients and Controls at different sessions, as assessed by the STAI.
**Figure S5:** 9/11 Episodic Question. A TEMPau‐graded episodic question scored over 10 points the participants' recall of what they were doing when the 9/11 events unfolded. Only time effect was statistically significant (Time: p = 0.001; Group: p = 0.18; Interaction: p = 0.34). Considering Day 0 results alone, there still was no statistically significant difference between groups (T = −0.99, p = 0.32).
**Figure S6:** Relation between structural lesions and neuropsychological performance. Barplots: neuropsychological performances in three different tests according to the number of hippocampal lesions. Boxplot: connectivity in lesioned vs. not unlesioned patients.
**Figure S7:** Connectivity of the anterior thalamic nuclei (ATN). In the extended hippocampal system, corresponding to a subnetwork of the EMN (bilateral hippocampi, parahippocampal gyrus, PCC) plus left and right ATN, the only significant effects were those found in the EMN for common nodes (bilateral PHG—PCC connections). Thus, no significant effect of connectivity between the ATNs and any of significant clusters of the EMN was found. For exploratory purposes, and because of this intriguing Day 0 profile, we present some ATN effects without multiple comparisons correction. Top row: There is a positive interaction effect (F(2,76) = 0.17, p = 0.04) in the connectivity of the left ATN to the rest of the extended hippocampal system (bilateral thalamus, PHG, hippocampus, PCC), with significantly superior connectivity in patients relative to controls at Day 0. Bottom row: the left ATN connexions showing any significant effect were with left and right hippocampus.
**Figure S8:** ICA maps.
**Figure S9:** ICA results. Each component's intrinsic connectivity is computed as component‐level Z‐score averages (across voxels), and plotted below. No significant group, time or interaction effect was found for any of these components (see Supplementary Statistics).
**Table S1:** Characteristics of sMRI lesions.

**Data S1:** Supporting Information.

**Data S2:** Supporting Information.

## Data Availability

Light data such as neuropsychological data, 1st‐level preprocessed fMRI ROI‐to‐ROI data and python codes are available at https://github.com/EliasElOtmani/TGA. Heavy data such as raw fMRI, ICA and MRI data are available upon request.
