# Supplementary material for: Memory and Resting‐State Connectivity in Acute Transient Global Amnesia: A Case–Control fMRI Study
Source: Ann Clin Transl Neurol. 2026 Apr 10;13(11):2234–44. doi: 10.1002/acn3.70396 (PMC13394162; doi:10.1002/acn3.70396)

Supplementary Data

Supp. Table 1 - Characteristics of sMRI lesions

Patient no.	Number of lesions	Hippocampus affected	Locaation of the lesions (x,y,z)	CA1-ExtraCA1	Size of the lesions (number of voxels)
1	4	pHipp L<br>pHipp L<br>pHipp R<br>pHipp R	(57, 73, 30)<br>(59, 65, 33)<br>(89, 75, 30)<br>(91, 71, 32)	CA1<br>CA1<br>CA1<br>CA1	2<br>6<br>4<br>5
2	2	pHipp L<br>pHipp R	(51, 63, 30)<br>(78, 60, 30)	CA1<br>CA1	7<br>3
3	1	pHipp L	(52, 61, 39)	Extra-CA1	4
4	1	pHipp L	(49, 63, 21)	CA1	8
5	2	aHipp L<br>aHipp R	(49, 73, 29)<br>(76, 72, 27)	Extra-CA1<br>Extra-CA1	5<br>6
6	1	pHipp R	(78, 59, 39)	Extra-CA1	6
7	1	aHipp R	(80, 71, 21)	CA1	10
8	1	pHipp L	(56, 70, 34)	CA1	3
9	1	pHipp R	(88, 67, 38)	CA1	6
10	1	pHipp L	(49, 60, 18)	CA1	1
11	3	pHipp L<br>aHipp L<br>pHipp R	(60, 66, 19)<br>(60, 75, 17)<br>(91, 75, 18)	CA1<br>CA1<br>CA1	4<br>5<br>3
12	1	pHipp L	(53, 55, 24)	CA1	4
13	0				
14	1	pHipp L	(50, 58, 22)	CA1	4
15	1	pHipp L	(48, 62, 37)	CA1	3
16	3	pHipp R<br>aHipp R<br>pHipp R	(80, 66, 35)<br>(88, 81, 25)<br>(90, 75, 27)	Extra-CA1<br>CA1<br>CA1	2<br>1<br>9
17	2	pHipp L<br>pHipp R	(49, 65, 32)<br>(80, 66, 34)	CA1<br>CA1	6<br>6
18	1	aHipp L	(51, 63, 27)	CA1	4
19	0				
20	2	pHipp L<br>pHipp R	(55, 55, 24)<br>(86, 61, 20)	CA1<br>CA1	6<br>5

Note that among the 7 patients having more than one lesion, we don't observe any two lesions landing in the same hippocampal subdivision within any patient. 6 out of these 7 patients have bilateral lesions, and out of the 15 corresponding lesions of these 6 patients, 14 are symetrical in terms of hippocampal subdivisions (head, body, tail ; CA1 or not CA1). This advocates for a hippocampally diffuse aetiology as opposed to a focal one.

Supplementary Figure 1 : Patients' flow diagram

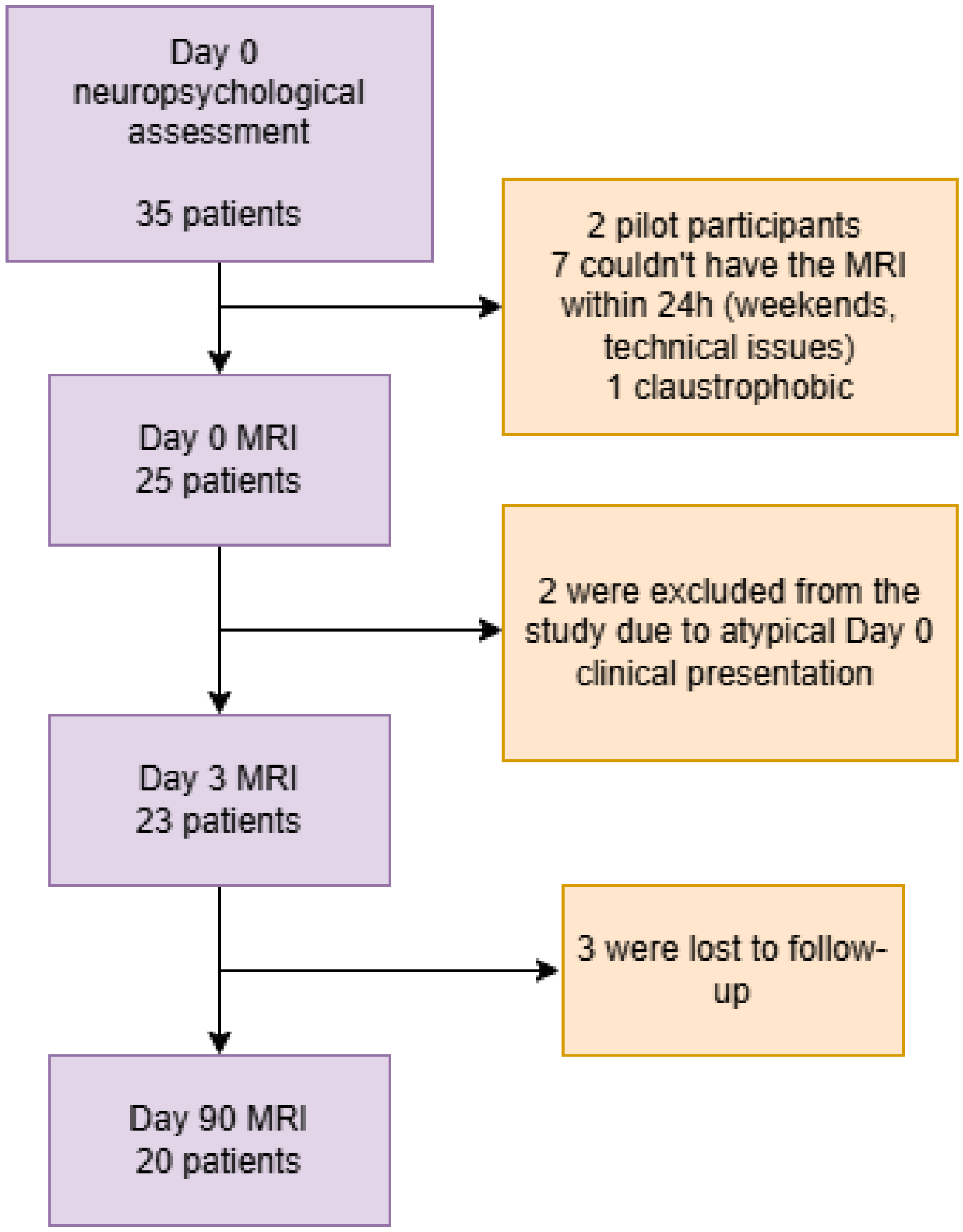

Supplementary Figure 2 : Total free recall, cued recall and recognition performances in Mnemosyne's episode 2 at day 0. This section of the Mnemosyne evaluation is a multimodal incidental encoding task

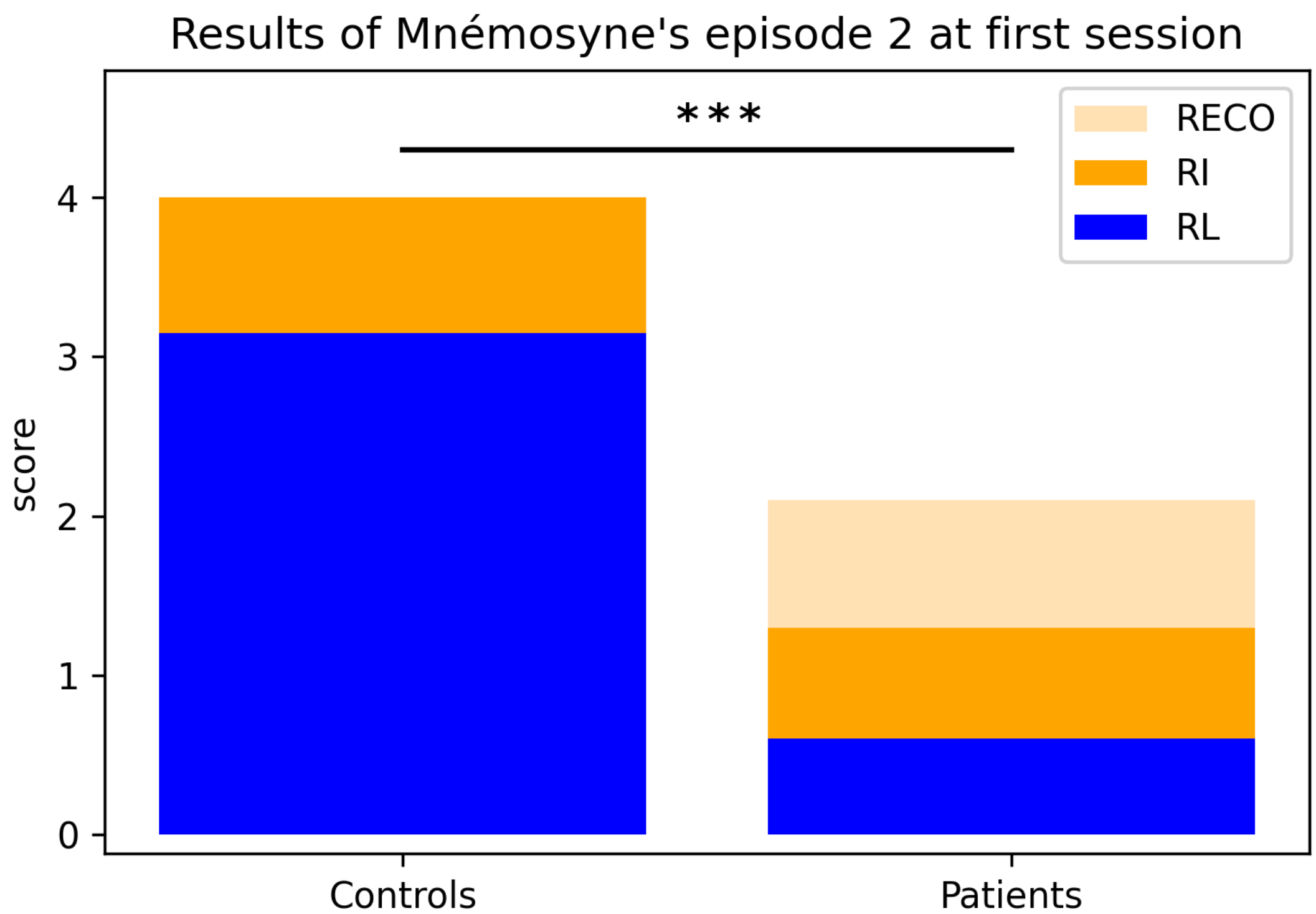

Supplementary Figure 3 : **Correlation matrix and clustering dendrogram of patient's performances in neuropsychological testing at Day 0**. "Who" and "What" refer to questions about the 9/11 attack, asking for recall of, respectively, a famous character involved and the reason for the attack. Other tests are detailed in the Methods section.

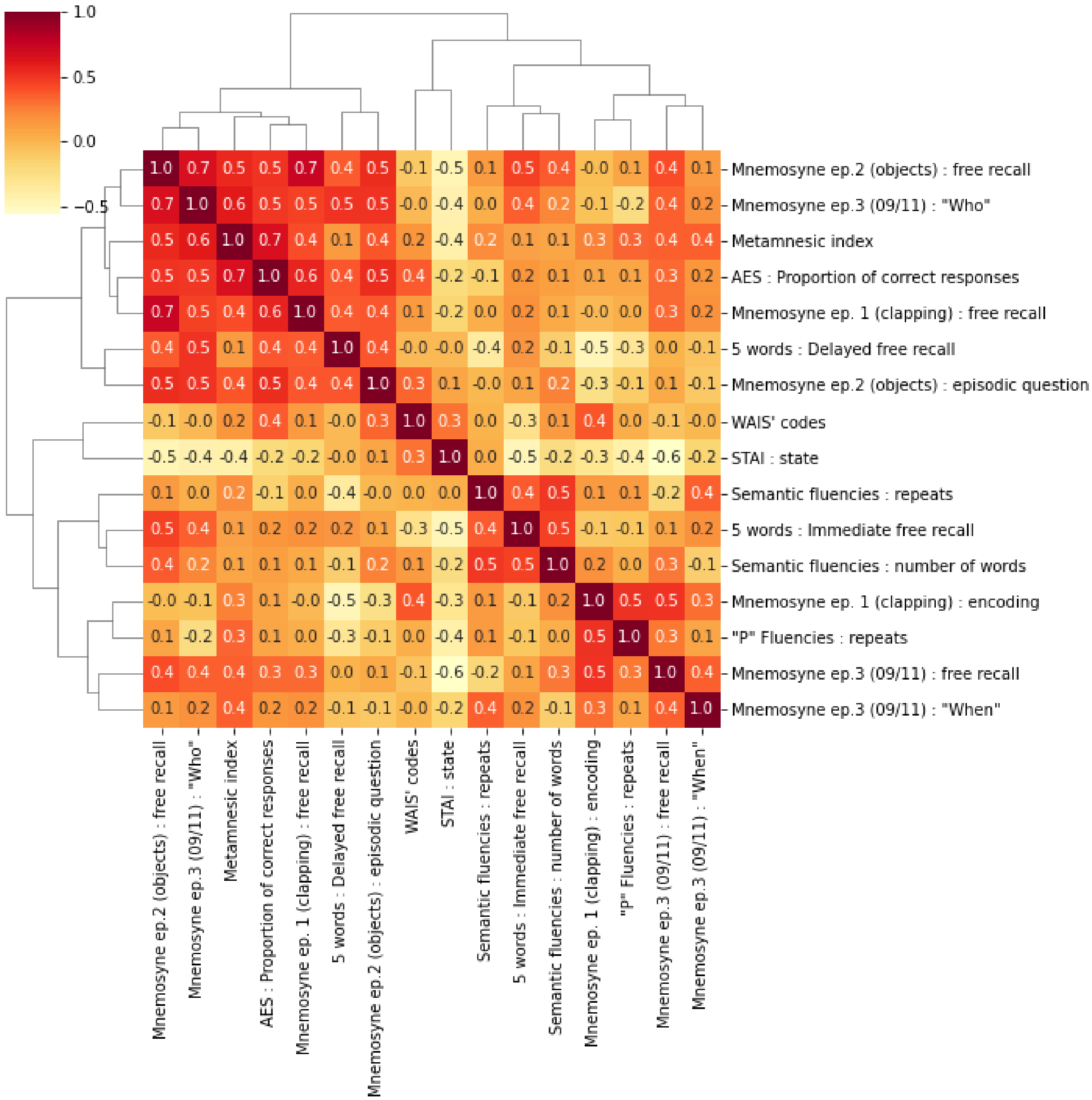

Supplementary Figure 4 : **state of anxiety between Patients and Controls at different sessions, as assessed by the STAI**

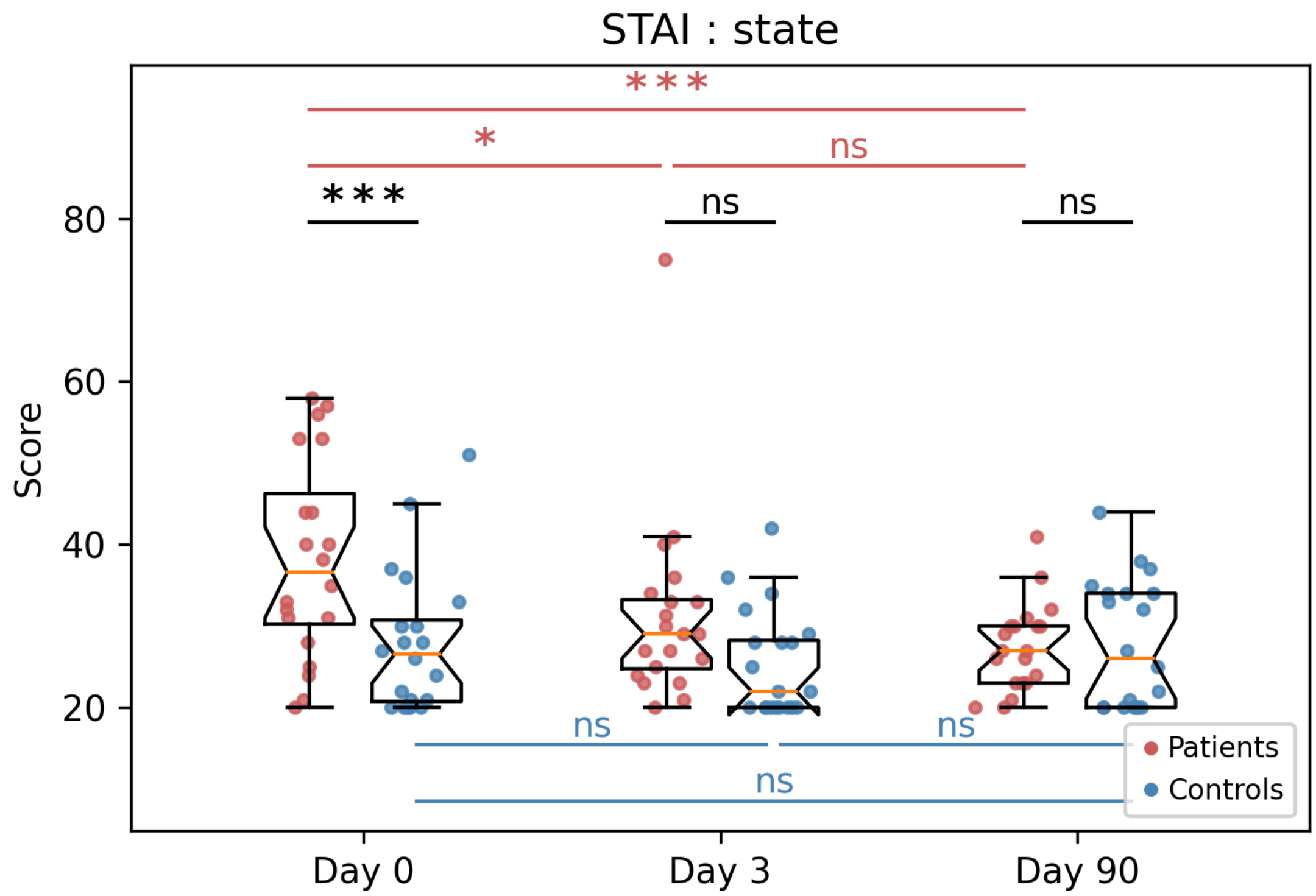

Supplementary Figure 5 : 9/11 Episodic Question. A TEMPau-graded episodic question scored over 10 points the participants' recall of what they were doing when the 9/11 events unfolded. Only time effect was statistically significant (Time : p = 0.001 ; Group : p = 0.18 ; Interaction : p = 0.34). Considering Day 0 results alone, there still was no statistically significant difference between groups (T = -0.99, p = 0.32).

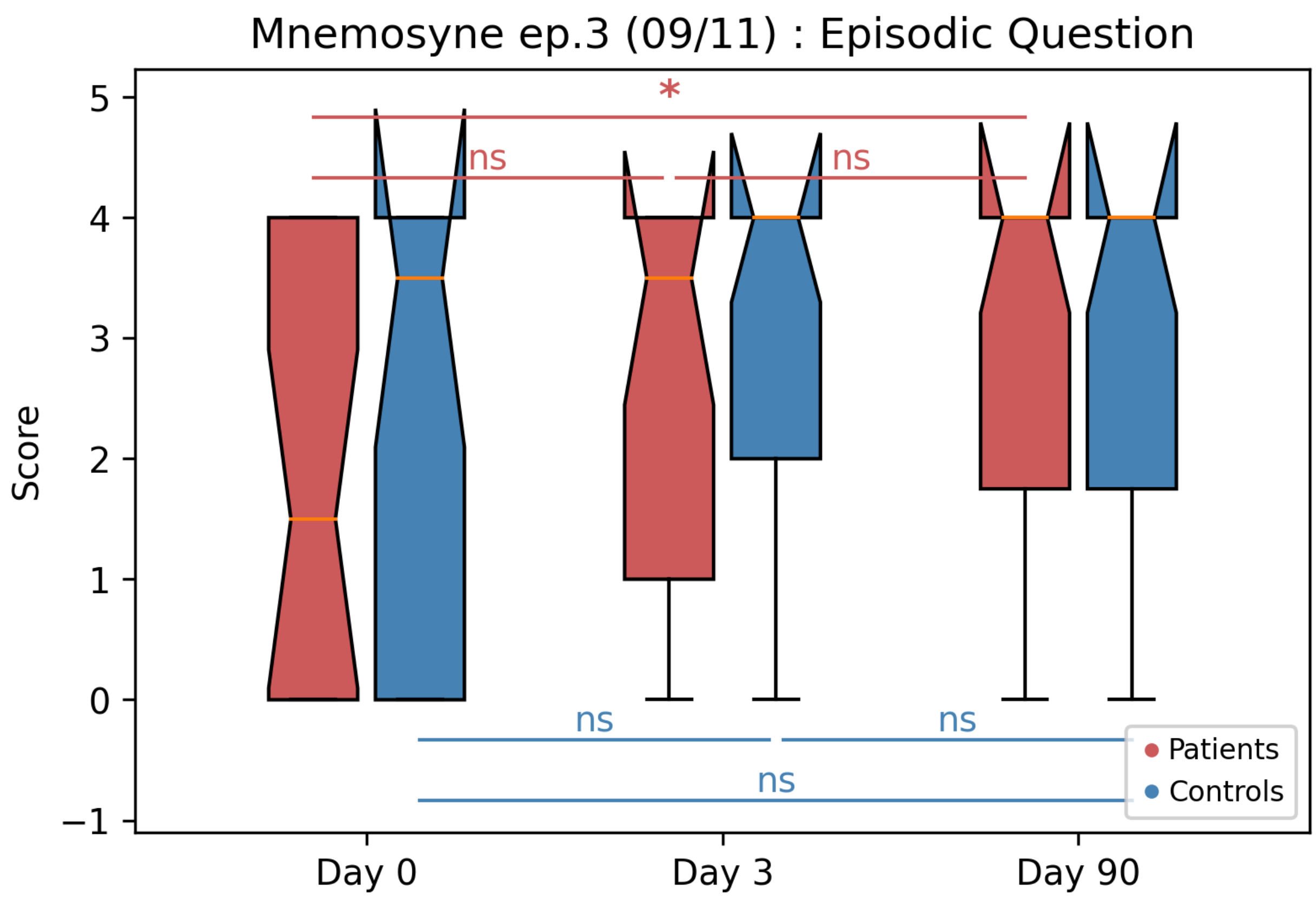

Supplementary Figure 6 : **Relation between structural lesions and neuropsychological performance**. Barplots : neuropsychological performances in three different tests according to the number of hippocampal lesions. Boxplot : connectivity in lesioned vs not unlesioned patients.

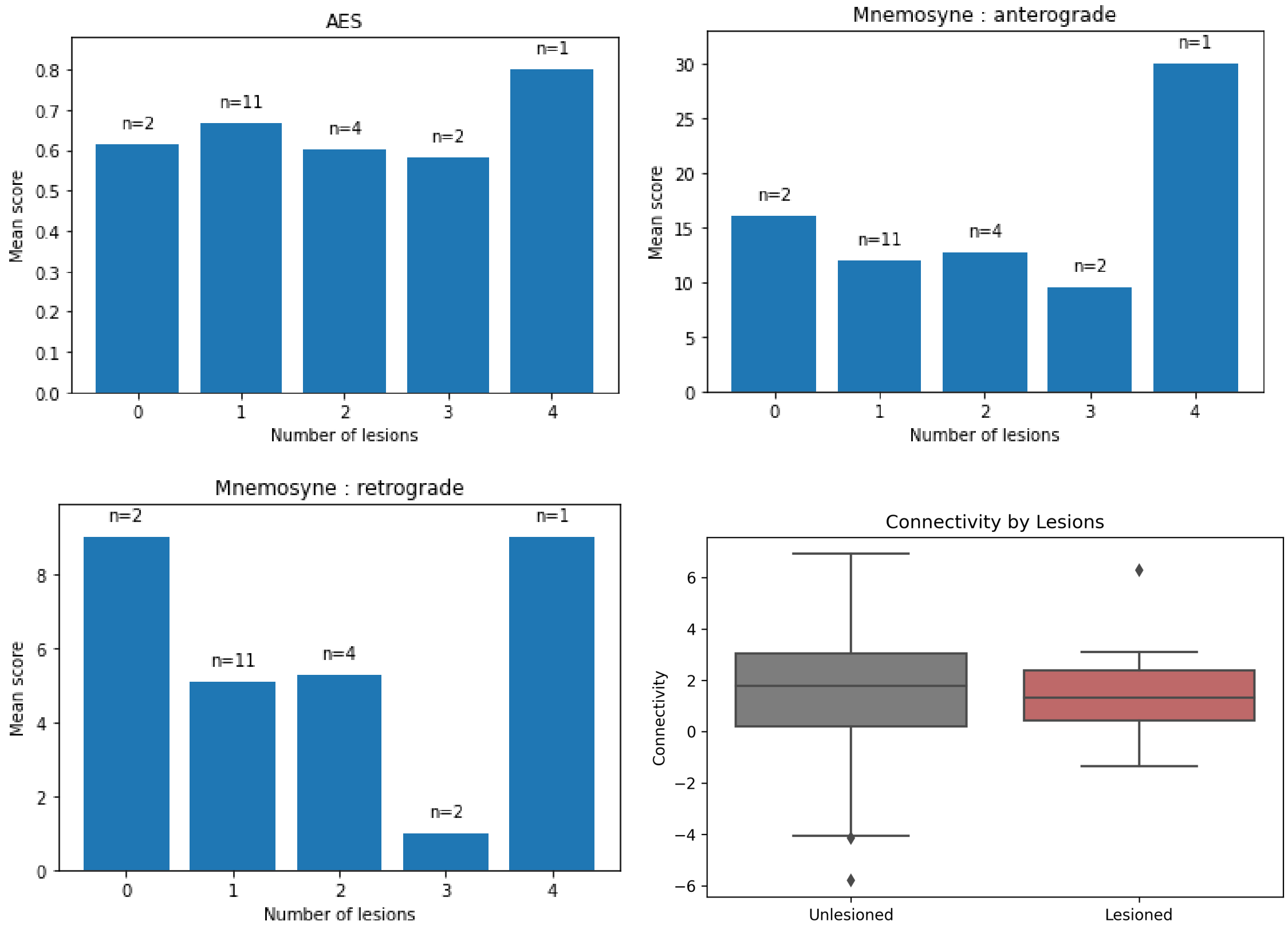

Supplementary Figure 7 : **Connectivity of the anterior thalamic nuclei (ATN)**.
In the extended hippocampal system, corresponding to a subnetwork of the EMN (bilateral hippocampi, parahippocampal gyrus, PCC) plus left and right ATN, the only significant effects were those found in the EMN for common nodes (bilateral PHG - PCC connections). Thus, no significant effect of connectivity between the ATNs and any of significant clusters of the EMN was found. For exploratory purposes, and because of this intriguing Day 0 profile, we present some ATN effects without multiple comparisons correction.
Top row : There is a positive interaction effect ($F(2,76) = 0.17$, $p = 0.04$) in the connectivity of the left ATN to the rest of the extended hippocampal system (bilateral thalamus, PHG, hippocampus, PCC), with significantly superior connectivity in patients relative to controls at Day 0. Bottom row : the left ATN connexions showing any significant effect were with left and right hippocampus.

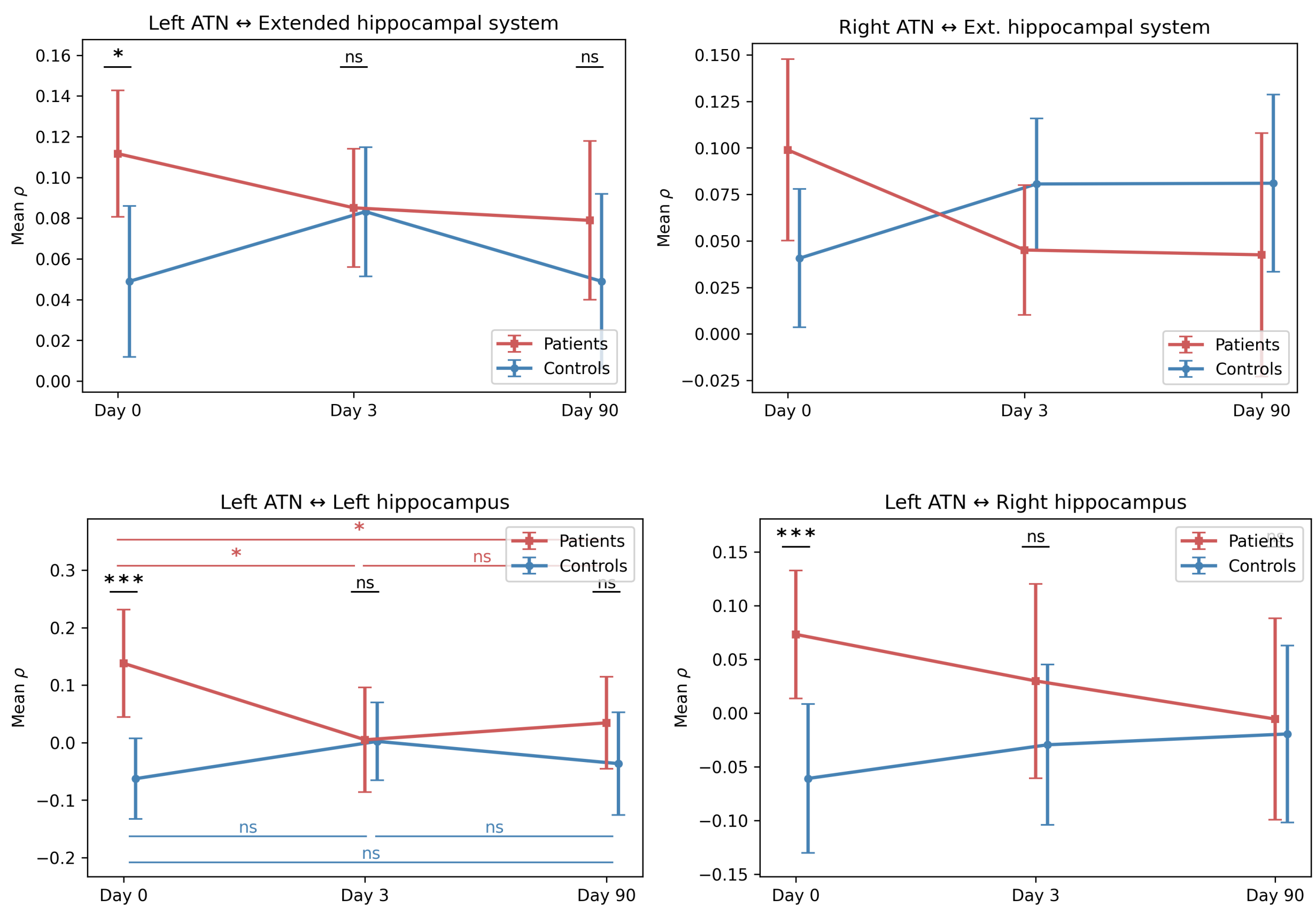

Supplementary Figure 8 : ICA maps

Spatial correlation coefficients are denoted “r”. Warm colors indicate voxels positively correlated with the component, cold colors indicate voxels negatively correlated with the component.

“**Ventral DMN**” (r=0.44)

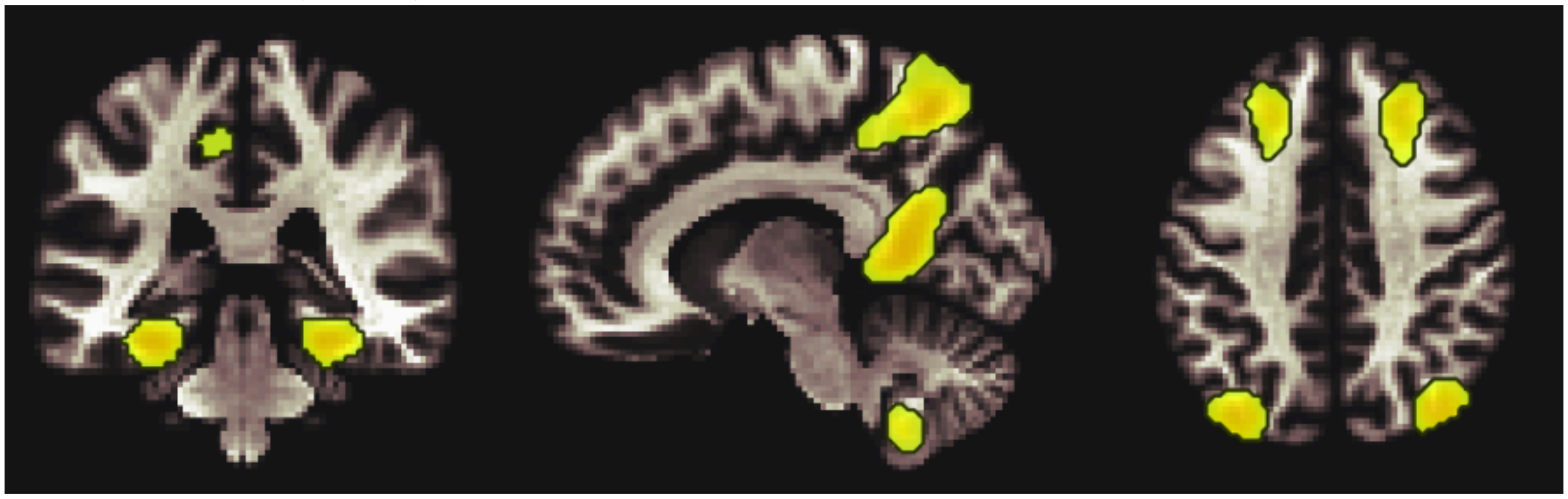

“**Dorsal DMN**” (r = 0.30)

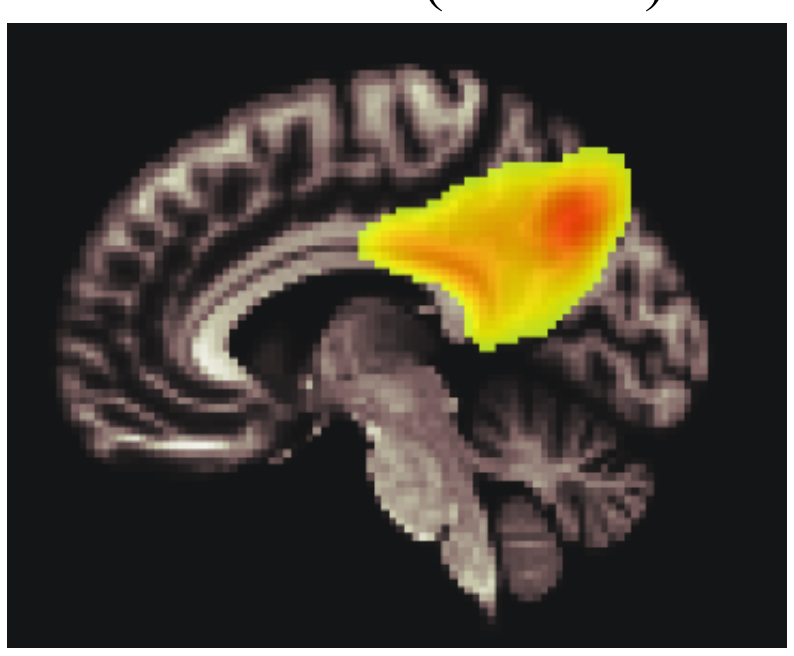

ECN (r = 0.42)

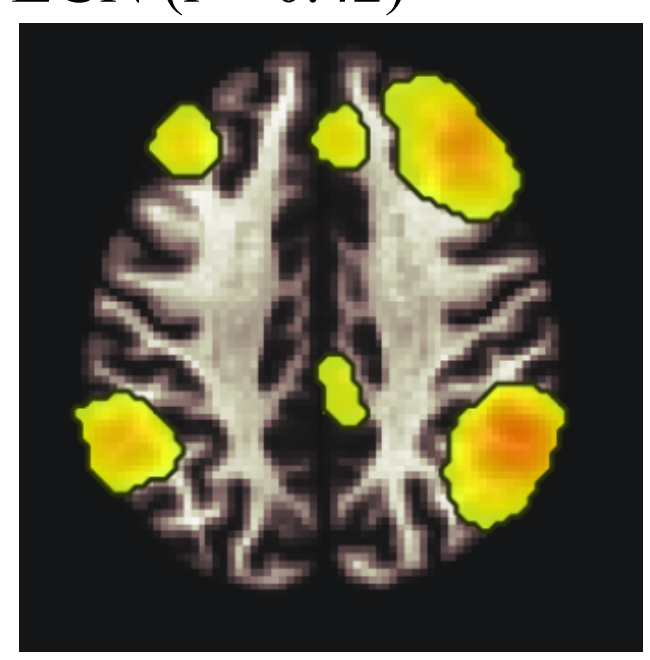

SN : 2 ICs interpreted as Left (r = 0.27, left images) and Right (r = 0.28, right image) SN. Blue zones denote negative loadings (voxels anticorrelated with the IC)

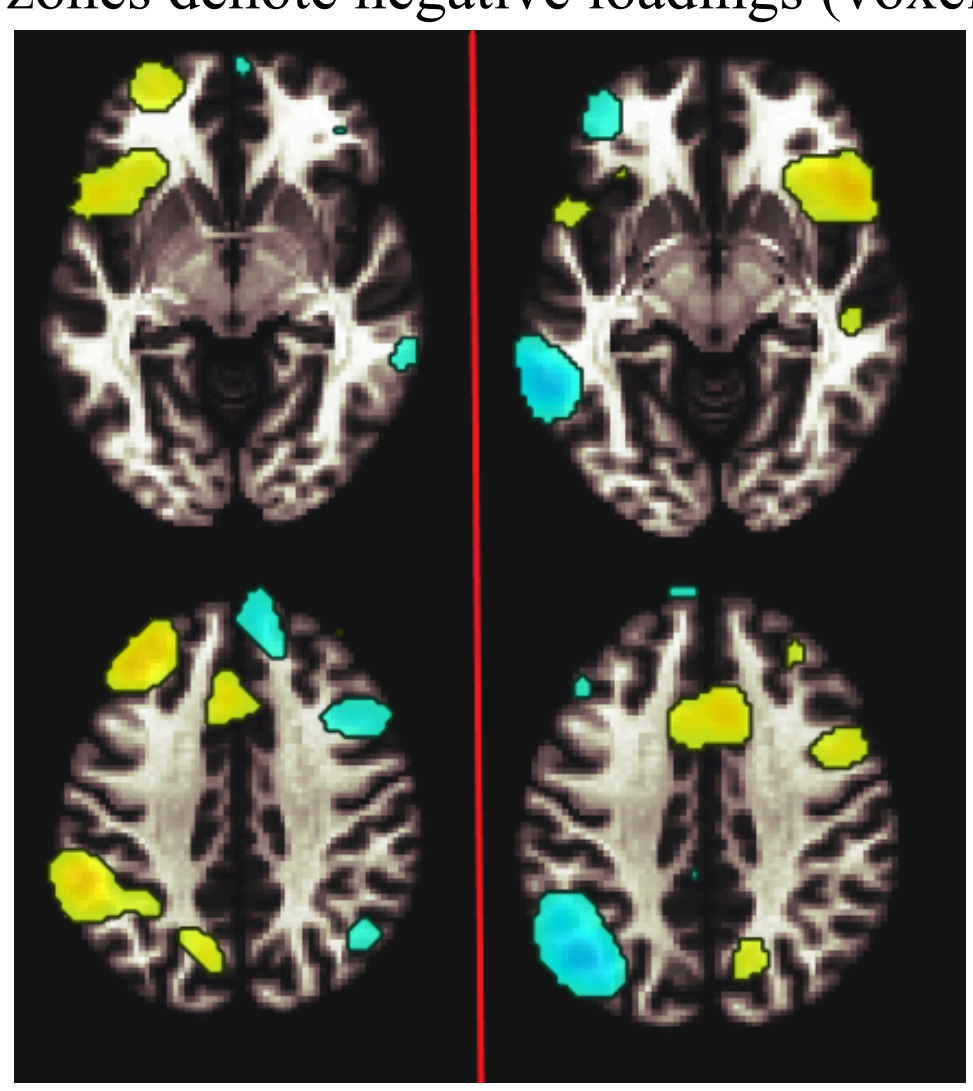

Supplementary Figure 9 : ICA results. Each component's intrinsic connectivity is computed as component-level Z-score averages (across voxels), and plotted below. No significant group, time or interaction effect was found for any of these components (see Supplementary Statistics).

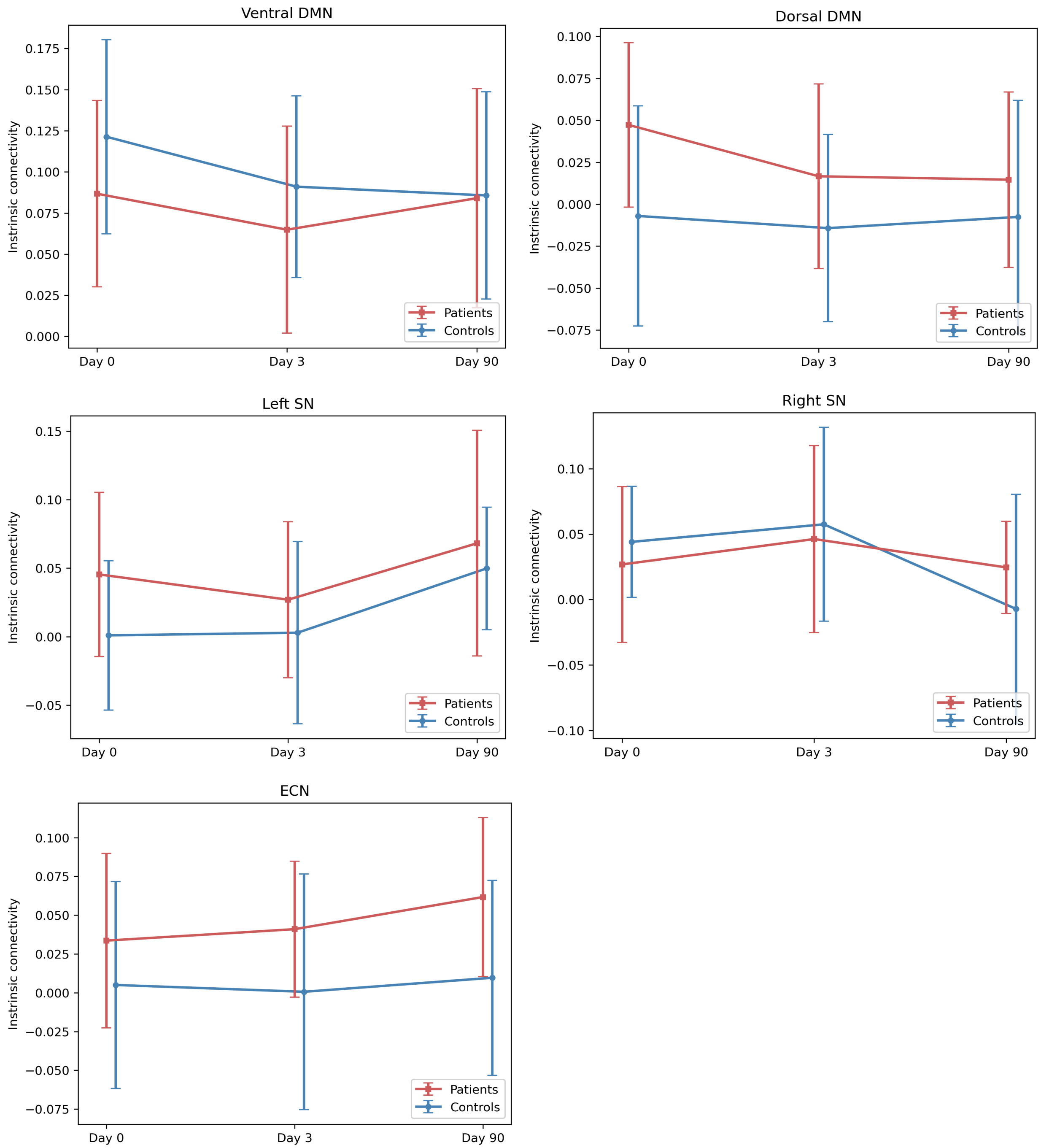

Supplement: Supplementary file 1 [file ACN3-13-2234-s001.pdf]
