# Supplementary material for: Memory and Resting‐State Connectivity in Acute Transient Global Amnesia: A Case–Control fMRI Study
Source: Ann Clin Transl Neurol. 2026 Apr 10:10.1002/acn3.70396. Online ahead of print. doi: 10.1002/acn3.70396 (PMC13394162; doi:10.1002/acn3.70396)
Supplement: Supplementary file 2 — Data S1: Supporting Information. [file ACN3-9999-0-s001.pdf]

Supplementary Methods

I - Recruitement & study design

1. Study design

We conducted a single-center, exploratory, prospective, controlled study at Toulouse University Hospital. The objective was to compare resting-state fMRI functional connectivity between a group of patients in the acute phase of transient global amnesia (TGA) and a group of healthy controls.

2. Study timeline

- Start of enrollment: November 2013
- Enrollment period: 24 months
- Duration of participation for each subject: 3 months
  The study was therefore expected to run until November 2015.

3. Participants

3.1 Inclusion criteria

Patient group.
Eligible patients were 40–80 years old and met the diagnostic criteria for TGA (Hodges & Warlow, 1990):

- acute anterograde memory disturbance observed by a witness,
- score ≤ 8/10 on the 5-word test (Dubois et al., 2002),
- and/or temporal-spatial orientation subscore of the MMSE ≤ 7/10 (Folstein et al., 1975),
- naming score > 9/10 on the first 10 items of the DO80 Oral Naming Test (Deloche et al., 1997),
- preservation of other cognitive functions on neurological examination.

In addition, patients had to show clinical resolution of the amnesic syndrome at 24 hours (reported by relatives) and confirmed at 72 hours (visit 2) by:

- absence of amnesic complaints from patient and relatives,
- 5-word test score ≥ 9/10,
- MMSE temporal-spatial orientation score ≥ 9/10.

Patients were initially included based on the clinical diagnosis of TGA at visit 1, and retained only if MRI and recovery criteria were met at visit 2.

Control group.
Controls had to meet both of the following:

- no cognitive complaints,
- no history of TGA.

3.2 Exclusion criteria

Patients.

- Any clinical signs inconsistent with TGA: altered consciousness or identity loss, seizure features (loss of contact, automatisms, abnormal movements), or focal neurological deficit.

Both groups.

- Recent head trauma (< 1 month),
- Acute alcohol or drug intoxication,
- Hypoglycemia (glucose < 0.5 g/L),
- Psychiatric disorder impairing judgment,
- Progressive neurological disease with cognitive impairment (e.g. Alzheimer's, advanced Parkinson's),
- Contraindications to MRI (pacemaker, cardiac defibrillator, electrically/magnetically activated implant, intracranial/carotid clips, orthopedic implants, claustrophobia),
- Insufficient fluency in French for neuropsychological testing,
- Administrative issues (inability to provide informed consent, lack of social security coverage, refusal to sign consent, legal guardianship),
- Pregnancy.

4. Ethics

The study was approved by the local ethics committee (CPP) on November 8, 2013 (protocol n°13 201 02, version 4, dated 28/10/2013). All data were collected in anonymized form.

As this was a biomedical study in an emergency setting, patients could be included upon arrival at the emergency department without prior consent. When possible, consent was obtained from a relative or accompanying person in accordance with French law (Article L.1122-1-2). In all cases, informed consent from the patient was systematically collected at 72 hours once recovery allowed.
Control subjects gave written informed consent at the first visit.

5. Study procedures

Participants underwent three MRI sessions and three neuropsychological assessments over a 3-month period. Each neuropsychological assessment lasted 30–45 minutes, and MRI 25

minutes. No contrast agent was administered. Control subjects received €150 compensation for the full study. All clinical MRI sequences (diffusion, T1, T2) were reviewed by Prof. Fabrice Bonneville (INSERM U825).

6. Recruitment

We aimed to include 20 patients and 20 controls over 2 years.

Patients.
Patients were recruited consecutively. An emergency pathway was established with the vascular neurology service (Prof. Chollet, Pierre-Paul Riquet Hospital, CHU Toulouse) to optimize recruitment and enable early examinations. Upon suspicion of TGA at triage, the on-call neurologist was notified and confirmed the diagnosis using clinical examination and a rapid test battery (5-word test, MMSE orientation subscore, DO80 naming items). Patients were then referred to the emergency outpatient unit and fully managed by a study investigator (eligibility verification, research procedures) until discharge or transfer.

This pathway operated 24/7 throughout the enrollment period. MRI was available Monday–Friday (9 a.m.–6 p.m.), with an emergency procedure in place to shorten delays.

Controls.
Healthy volunteers were recruited through posters and matched one-to-one with patients for age, sex, and education.

7. Note on data collection and analysis periods

We acknowledge that data acquisition occurred several years before the present analysis. The data were collected as part of an earlier project whose initial analyses were not pursued to publication. The present study constitutes a renewed analysis of these data using updated methodological and theoretical frameworks, allowing for a more accurate characterization of the network mechanisms involved.

II - MRI aquisition and analysis

Results included in this manuscript come from analyses performed using CONN[1] (RRID:SCR_009550) release 18.b[2] and SPM[3] (RRID:SCR_007037) release 12.7771.

MRI aquisition

We used an Achieva 3T scanner (Philips, Best, The Netherlands). 3D T1- and T2-weighted anatomical images were acquired to aid spatial normalisation. Diffusion-weighted images (DWI) in the axial and coronal axes were acquired (b = 2 000 s/mm2, 2 × 2 × 3 mm resolution, 3-mm slice thickness, 124 × 128 acquisition matrix. rsfMRI was performed with whole-brain, gradient-echo echoplanar imaging (EPI) T2*, with a 4 x 4 x 3 mm3 voxel size. Forty-six slices were acquired in each sequence. Participants were instructed to stay awake, with their eyes open, during the acquisition time (9 min 38 s). The total MRI session lasted 25 minutes.

Preprocessing: Functional and anatomical data were preprocessed using a flexible preprocessing pipeline[4] including realignment with correction of susceptibility distortion interactions, slice timing correction, outlier detection, direct segmentation and MNI-space normalization, and smoothing. Functional data were realigned using SPM realign & unwarp procedure[5], where all scans were coregistered to a reference image (first scan of the first session) using a least squares approach and a 6 parameter (rigid body) transformation[6], and resampled using b-spline interpolation to correct for motion and magnetic susceptibility interactions. Temporal misalignment between different slices of the functional data was corrected following SPM slice-timing correction (STC) procedure[7,8], using sinc temporal interpolation to resample each slice BOLD timeseries to a common mid-acquisition time. Potential outlier scans were identified using ART[9] as acquisitions with framewise displacement above 0.9 mm or global BOLD signal changes above 5 standard deviations[10,11], and a reference BOLD image was computed for each subject by averaging all scans excluding outliers. Functional and anatomical data were normalized into standard MNI space, segmented into grey matter, white matter, and CSF tissue classes, and resampled to 2 mm isotropic voxels following a direct normalization procedure[11,12] using SPM unified segmentation and normalization algorithm[13,14] with the default IXI-549 tissue probability map template. Last, functional data were smoothed using spatial convolution with a Gaussian kernel of 8 mm full width half maximum (FWHM).

Denoising: In addition, functional data were denoised using a standard denoising pipeline[15] including the regression of potential confounding effects characterized by white matter timeseries (5 CompCor noise components), CSF timeseries (5 CompCor noise components), session and task effects and their first order derivatives (6 factors), and linear trends (2 factors) within each functional run, followed by bandpass frequency filtering of the BOLD timeseries[16] between 0.008 Hz and 0.09 Hz. CompCor[17,18] noise components within white matter and CSF were estimated by computing the average BOLD signal as well as the largest principal components orthogonal to the BOLD average within each subject's eroded segmentation masks. From the number of noise terms included in this denoising strategy, the effective degrees of freedom of the BOLD signal after denoising were estimated to range from 248.5 to 250.3 (average 248.5) across all subjects[11].

Extended Hippocampal System definition :

The anterior thalamic nuclei (ATN) weren't included in the version of the EMN we investigated. Given their importance in anterograde memory, we also carried a supplementary analysis on most of the extended hippocampal system (see Discussion), composed of bilateral ATN and of the mesial subnetwork of the EMN : bilateral PHG, hippocampus and PCC. We did not study the mamillary bodies as they weren't included in the AAL atlas we used.

First-level and group-level analysis

Episodic Memory Network (and Extended Hippocampal System) :

First-level analysis ROI-to-ROI connectivity (RRC) matrices were estimated characterizing the functional connectivity between each pair of regions among 15 ROIs. Functional connectivity strength was represented by Fisher-transformed bivariate correlation coefficients from a general linear model (weighted-GLM[19]), estimated separately for each pair of ROIs, characterizing the association between their BOLD signal timeseries. Individual scans were weighted by a boxcar signal characterizing each individual task or experimental condition convolved with an SPM canonical hemodynamic response function and rectified.

In EMN second-level analyses, each ROI was treated as a cluster (all its connections), with a separate GLM[39] estimating connection-level connectivities as dependent variables and group/session as predictors. ROI-level hypotheses were tested using multivariate parametric statistics with random-effects across subjects and covariance estimation across repeated measures. Inferences were made at the cluster level with familywise correction ($p\text{-FWE} < 0.05$). For significant clusters, connection-level GLMs were then fitted to identify the connections driving the effect; post-hoc significance was defined as uncorrected $p < 0.01$.

Each participant's whole-network EMN connectivity was the mean of all its connectivity values. It was analyzed through mixed model ANOVA.

DMN, ECN, SN

Unlike EMN, DMN, ECN and SN are major resting state networks, better suited for analysis through Independent Component Analysis (ICA).

First-level analysis ICA: Group-level ICA (group-ICA[24]) were performed to estimate 40 temporally coherent networks from the fMRI data combined across all subjects and conditions. The BOLD signal from every timepoint and voxel in the brain was concatenated across subjects and conditions along the temporal dimension. A singular value decomposition of the z-score normalized BOLD signal (subject-level SVD) with 64 components separately for each subject and condition was used as a subject-specific dimensionality reduction step. The dimensionality of the concatenated data was further reduced using a singular value decomposition (group-level SVD) with 40 components, and a fast-ICA fixed-point algorithm[25] with gaussian (G2) contrast function was used to identify spatially independent group-level networks from the resulting components. Last, GICA1 back-projection[26] was used to compute ICA maps associated with these same networks separately for each individual subject and condition.

Group-level analyses were performed using a GLM. For each individual voxel a separate GLM was estimated, with first-level connectivity measures at this voxel as dependent variables (one independent sample per subject and one measurement per session), and groups and sessions as independent variables. Voxel-level hypotheses were evaluated using multivariate parametric statistics with random-effects across subjects and sample covariance estimation across multiple measurements. Inferences were performed at the level of individual clusters (groups of contiguous voxels). Cluster-level inferences were based on parametric statistics from Gaussian Random Field theory[27,28]. Results were thresholded using a combination of a cluster-forming $p < 0.001$ voxel-level threshold, and a familywise corrected p-FDR < 0.05 cluster-size threshold[29].

References

[1] Whitfield-Gabrieli, S., & Nieto-Castanon, A. (2012). Conn: a functional connectivity toolbox for correlated and anticorrelated brain networks. Brain connectivity, 2(3), 125-141.

[2] Nieto-Castanon, A. & Whitfield-Gabrieli, S. (2018). CONN functional connectivity toolbox: RRID SCR_009550, release 18. doi:10.56441/hilbertpress.1818.9585.

[3] Penny, W. D., Friston, K. J., Ashburner, J. T., Kiebel, S. J., & Nichols, T. E. (Eds.). (2011). Statistical parametric mapping: the analysis of functional brain images. Elsevier.

[4] Nieto-Castanon, A. (2020). FMRI minimal preprocessing pipeline. In Handbook of functional connectivity Magnetic Resonance Imaging methods in CONN (pp. 3–16). Hilbert Press.

[5] Andersson, J. L., Hutton, C., Ashburner, J., Turner, R., & Friston, K. J. (2001). Modeling geometric deformations in EPI time series. Neuroimage, 13(5), 903-919.

[6] Friston, K. J., Ashburner, J., Frith, C. D., Poline, J. B., Heather, J. D., & Frackowiak, R. S. (1995). Spatial registration and normalization of images. Human brain mapping, 3(3), 165-189.

[7] Henson, R. N. A., Buechel, C., Josephs, O., & Friston, K. J. (1999). The slice-timing problem in event-related fMRI. NeuroImage, 9, 125.

[8] Sladky, R., Friston, K. J., Tröstl, J., Cunnington, R., Moser, E., & Windischberger, C. (2011). Slice-timing effects and their correction in functional MRI. Neuroimage, 58(2), 588-594.

[9] Whitfield-Gabrieli, S., Nieto-Castanon, A., & Ghosh, S. (2011). Artifact detection tools (ART). Cambridge, MA. Release Version, 7(19), 11.

[10] Power, J. D., Mitra, A., Laumann, T. O., Snyder, A. Z., Schlaggar, B. L., & Petersen, S. E. (2014). Methods to detect, characterize, and remove motion artifact in resting state fMRI. Neuroimage, 84, 320-341.

[11] Nieto-Castanon, A. (submitted). Preparing fMRI Data for Statistical Analysis. In M. Filippi (Ed.). fMRI techniques and protocols. Springer. doi:10.48550/arXiv.2210.13564

[12] Calhoun, V.D., Wager, T.D., Krishnan, A., Rosch, K.S., Seymour, K.E., Nebel, M.B., Mostofsky, S.H., Nyalakanai, P. and Kiehl, K. (2017). The impact of T1 versus EPI spatial normalization templates for fMRI data analyses (Vol. 38, No. 11, pp. 5331-5342).

[13] Ashburner, J., & Friston, K. J. (2005). Unified segmentation. Neuroimage, 26(3), 839-851.

[14] Ashburner, J. (2007). A fast diffeomorphic image registration algorithm. Neuroimage, 38(1), 95-113.

[15] Nieto-Castanon, A. (2020). FMRI denoising pipeline. In Handbook of functional connectivity Magnetic Resonance Imaging methods in CONN (pp. 17–25). Hilbert Press.

[16] Hallquist, M. N., Hwang, K., & Luna, B. (2013). The nuisance of nuisance regression: spectral misspecification in a common approach to resting-state fMRI preprocessing reintroduces noise and obscures functional connectivity. Neuroimage, 82, 208-225.

[17] Behzadi, Y., Restom, K., Liau, J., & Liu, T. T. (2007). A component based noise correction method (CompCor) for BOLD and perfusion based fMRI. Neuroimage, 37(1), 90-101.

[18] Chai, X. J., Nieto-Castanon, A., Ongur, D., & Whitfield-Gabrieli, S. (2012). Anticorrelations in resting state networks without global signal regression. Neuroimage, 59(2), 1420-1428.

[19] Nieto-Castanon, A. (2020). Functional Connectivity measures. In Handbook of functional connectivity Magnetic Resonance Imaging methods in CONN (pp. 26–62). Hilbert Press.

[20] Nieto-Castanon, A. (2020). General Linear Model. In Handbook of functional connectivity Magnetic Resonance Imaging methods in CONN (pp. 63–82). Hilbert Press.

[21] Zalesky, A., Fornito, A., & Bullmore, E. T. (2010). Network-based statistic: identifying differences in brain networks. Neuroimage, 53(4), 1197-1207.

[22] Nieto-Castanon, A. (2020). Cluster-level inferences. In Handbook of functional connectivity Magnetic Resonance Imaging methods in CONN (pp. 83–104). Hilbert Press.

[23] Benjamini, Y., & Hochberg, Y. (1995). Controlling the false discovery rate: a practical and powerful approach to multiple testing. Journal of the Royal statistical society: series B (Methodological), 57(1), 289-300.

[24] Calhoun, V. D., Adali, T., Pearlson, G. D., & Pekar, J. J. (2001). A method for making group inferences from functional MRI data using independent component analysis. Human brain mapping, 14(3), 140-151.

[25] Hyvarinen, A. (1999). Fast and robust fixed-point algorithms for independent component analysis. IEEE transactions on Neural Networks, 10(3), 626-634.

[26] Erhardt, E. B., Rachakonda, S., Bedrick, E. J., Allen, E. A., Adali, T., & Calhoun, V. D. (2011). Comparison of multi‐subject ICA methods for analysis of fMRI data. Human brain mapping, 32(12), 2075-2095.

[27] Worsley, K. J., Marrett, S., Neelin, P., Vandal, A. C., Friston, K. J., & Evans, A. C. (1996). A unified statistical approach for determining significant signals in images of cerebral activation. Human brain mapping, 4(1), 58-73.

[28] Nieto-Castanon, A. (2020). Cluster-level inferences. In Handbook of functional connectivity Magnetic Resonance Imaging methods in CONN (pp. 83–104). Hilbert Press.

[29] Chumbley, J., Worsley, K., Flandin, G., & Friston, K. (2010). Topological FDR for neuroimaging. Neuroimage, 49(4), 3057-3064.
